# Genetic modifiers in carriers of repeat expansions in the *C9ORF72* gene

**DOI:** 10.1186/1750-1326-9-38

**Published:** 2014-09-20

**Authors:** Marka van Blitterswijk, Bianca Mullen, Aleksandra Wojtas, Michael G Heckman, Nancy N Diehl, Matthew C Baker, Mariely DeJesus-Hernandez, Patricia H Brown, Melissa E Murray, Ging-Yuek R Hsiung, Heather Stewart, Anna M Karydas, Elizabeth Finger, Andrew Kertesz, Eileen H Bigio, Sandra Weintraub, Marsel Mesulam, Kimmo J Hatanpaa, Charles L White, Manuela Neumann, Michael J Strong, Thomas G Beach, Zbigniew K Wszolek, Carol Lippa, Richard Caselli, Leonard Petrucelli, Keith A Josephs, Joseph E Parisi, David S Knopman, Ronald C Petersen, Ian R Mackenzie, William W Seeley, Lea T Grinberg, Bruce L Miller, Kevin B Boylan, Neill R Graff-Radford, Bradley F Boeve, Dennis W Dickson, Rosa Rademakers

**Affiliations:** Department of Neuroscience, Mayo Clinic, 4500 San Pablo Road, Jacksonville, FL 32224 USA; Section of Biostatistics, Mayo Clinic, 4500 San Pablo Road, Jacksonville, FL 32224 USA; Division of Neurology, University of British Columbia, 2211 Wesbrook Mall, Vancouver, BC V6T 2B5 Canada; Department of Neurology, University of California, 500 Parnassus Ave, San Francisco, CA 94143 USA; The University of Western Ontario, 1151 Richmond St, London, ON N6A 3 K7 Canada; Cognitive Neurology and Alzheimer’s Disease Center, Northwestern University Feinberg School of Medicine, 320 East Superior Street, Chicago, IL 60611 USA; University of Texas Southwestern Medical Center, 5323 Harry Hines Blvd, Dallas, TX 75390 USA; Department of Neuropathology, University of Tübingen and German Center for Neurodegenerative Diseases, Calwerstr. 3, Tübingen, 72076 Germany; Molecular Brain Research Group, Robarts Research Institute, 100 Perth Drive, London, ON N6A 5 K8 Canada; Banner Sun Health Research Institute, 10515 W Santa Fe Dr, Sun City, AZ 85351 USA; Department of Neurology, Mayo Clinic, 4500 San Pablo Road, Jacksonville, FL 32224 USA; Department of Neurology, Drexel University College of Medicine, 2900 W Queen Ln, Philadelphia, PA 19129 USA; Department of Neurology, Mayo Clinic, 5777 E Mayo Blvd, Phoenix, AZ 85054 USA; Department of Neurology, Mayo Clinic, 1216 2nd St SW, Rochester, MN 55902 USA; Department of Pathology and Laboratory Medicine, University of British Columbia, 2329W Mall, Vancouver, BC V6T 1Z4 Canada

**Keywords:** C9ORF72, Frontotemporal dementia, Motor neuron disease, Genetic modifier, Repeat expansion

## Abstract

**Background:**

Hexanucleotide repeat expansions in chromosome 9 open reading frame 72 (*C9ORF72*) are causative for frontotemporal dementia (FTD) and motor neuron disease (MND). Substantial phenotypic heterogeneity has been described in patients with these expansions. We set out to identify genetic modifiers of disease risk, age at onset, and survival after onset that may contribute to this clinical variability.

**Results:**

We examined a cohort of 330 *C9ORF72* expansion carriers and 374 controls. In these individuals, we assessed variants previously implicated in FTD and/or MND; 36 variants were included in our analysis. After adjustment for multiple testing, our analysis revealed three variants significantly associated with age at onset (rs7018487 [*UBAP1*; p-value = 0.003], rs6052771 [*PRNP*; p-value = 0.003], and rs7403881 [*MT-Ie*; p-value = 0.003]), and six variants significantly associated with survival after onset (rs5848 [*GRN*; p-value = 0.001], rs7403881 [*MT-Ie*; p-value = 0.001], rs13268953 [*ELP3*; p-value = 0.003], the epsilon 4 allele [*APOE*; p-value = 0.004], rs12608932 [*UNC13A*; p-value = 0.003], and rs1800435 [*ALAD*; p-value = 0.003]).

**Conclusions:**

Variants identified through this study were previously reported to be involved in FTD and/or MND, but we are the first to describe their effects as potential disease modifiers in the presence of a clear pathogenic mutation (i.e. *C9ORF72* repeat expansion). Although validation of our findings is necessary, these variants highlight the importance of protein degradation, antioxidant defense and RNA-processing pathways, and additionally, they are promising targets for the development of therapeutic strategies and prognostic tests.

**Electronic supplementary material:**

The online version of this article (doi:10.1186/1750-1326-9-38) contains supplementary material, which is available to authorized users.

## Background

Two fatal neurodegenerative diseases, frontotemporal dementia (FTD) and motor neuron disease (MND), demonstrate clinical, pathological and genetic overlap. In up to 50% of FTD patients, for instance, signs of motor neuron dysfunction are present and an equal percentage of MND patients can show cognitive symptoms of frontal lobe impairment [1–4]. Moreover, inclusions of transactive response DNA-binding protein 43 (TDP-43) are the most common subtype of FTD and are also a pathological hallmark of MND [5, 6]. Interestingly, hexanucleotide repeat expansions in the chromosome 9 open reading frame 72 (*C9ORF72*) gene have been identified in FTD and MND [7, 8], representing the most frequent genetic cause of both diseases [9]. Considerable clinical variability, however, has been detected in carriers of these expansions, including heterogeneity in age at onset and disease duration [10]. While recent studies implicated variants in transmembrane protein 106 B (*TMEM106B*), intermediate repeats in ataxin-2 (*ATXN2*), *C9ORF72* expansion size, and the presence of double mutations as genetic modifiers of the clinical presentation in *C9ORF72* expansion carriers [11–15], it remains largely unknown why some individuals develop disease symptoms in their 40s whereas others remain unaffected until old age.

In *C9ORF72* expansion carriers, FTD and/or MND-associated variants that modify disease risk, age at onset or survival after onset have not been studied systematically. For this reason, we conducted a thorough literature search and included 36 known variants in our study. These variants were investigated in a cohort of 330 *C9ORF72* expansion carriers and 374 controls; importantly, we identified eight potential disease modifiers that may aid in explaining the reported phenotypic heterogeneity.

## Results

We investigated a cohort of 330 *C9ORF72* expansion carriers and 374 controls for 36 variants known to modify disease risk, age at onset or survival after onset in FTD and/or MND (Table 1; Additional file 1: Table S1). For simplicity, we have included an overview of significant associations, displaying only the genotypic model for which evidence of association was strongest (Table 2); results of all genotypic models for analyses that contained significant associations are shown in the supplement (Additional file 1: Table S2 [age at onset] and Additional file 1: Table S3 [survival after onset]).

**Table 1:** **Subject characteristics**

Group	N	Female gender	Age	Age at onset	Pathological diagnosis
Controls	374	172 (46.0%)	61.2 ± 10.2 (35–90)	N/A	N/A
All repeat expansion carriers	330	149 (45.2%)	59.4 ± 10.0 (35–90)	56.5 ± 9.1 (34–83)	123 (37.3%)
FTD, FTD/MND, and MND probands	265	115 (43.4%)	59.6 ± 10.0 (35–90)	56.8 ± 9.1 (34–83)	112 (42.3%)
FTD probands	74	29 (39.2%)	63.1 ± 12.2 (35–90)	57.7 ± 9.8 (34–79)	45 (60.8%)
FTD/MND probands	71	25 (35.2%)	60.6 ± 8.5 (39–80)	56.2 ± 9.0 (34–74)	51 (71.8%)
MND probands	120	61 (50.8%)	56.9 ± 8.6 (37–83)	56.5 ± 8.7 (36–83)	16 (13.3%)

Continuous variables are summarized with the sample mean ± standard deviation (range). The age provided is age at blood draw in controls, age at onset in clinically diagnosed patients, and age at death in pathologically diagnosed patients. Information was unavailable for age (n = 41) and age at onset (n = 59).

**Table 2:** **Variants significantly associated with age at onset or survival after onset**

Variant (gene/disease group)	Number of patients with each genotype ^a^	Model	Association measure (95% CI)	P-value
Age at onset (overall)			Regression coefficient	
rs7018487 (*UBAP1*)	122 / 96 / 23	Additive	−2.62 (−4.36, −0.89)	0.003
rs6052771 (*PRNP*)	92 / 104 / 46	Recessive	4.42 (1.51, 7.32)	0.003
rs7403881 (*MT-Ie*)	65 / 118 / 60	Dominant	3.95 (1.36, 6.54)	0.003
Survival after onset (overall)			Relative risk	
rs5848 (*GRN*)	116 / 86 / 19	Additive	1.64 (1.22, 2.22)	0.001
Survival after onset (disease subgroups)			Relative risk	
rs7403881 (*MT-Ie*): FTD	13 / 31 / 14	Recessive^b^	3.81 (1.71, 8.46)	0.001
rs13268953 (*ELP3*): FTD	16 / 31 / 11	Recessive	3.65 (1.56, 8.55)	0.003
Epsilon 4 (*APOE*): FTD	42 / 13 / 2	Dominant	3.13 (1.45, 6.74)	0.004
rs12608932 (*UNC13A*): MND	44 / 40 / 23	Recessive^b^	5.65 (1.82, 17.58)	0.003
rs1800435 (*ALAD*): MND	88 / 18 / 1	Dominant	N/A^c^	0.003

Association measure = regression coefficient (age at onset analysis) and relative risk (survival after onset analysis); CI = confidence interval. Additive models, dominant models, and recessive models were utilized. We adjusted for multiple testing using a false discovery rate (FDR) of 10%. ^a^Order of genotypes: major-major/major-minor/minor-minor. ^b^Indicates that the variant was also significantly associated with the given outcome under an additive model. ^c^For rs1800435, none of the 19 MND patients (0.0%) who carried the minor allele died as compared to 14 of 78 MND patients (15.9%) who did not carry the minor allele; the p-value of 0.003 results from a log-rank test. The strongest association with the given outcome is displayed in this table; other associations are shown in Additional file 1: Table S2 (age at onset) and Additional file 1: Table S3 (survival after onset).

**Figure 1:**
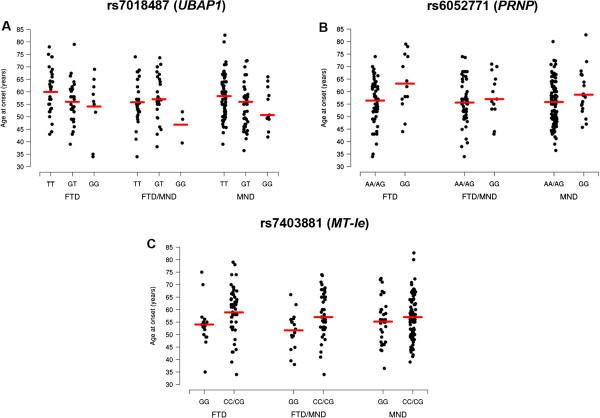
**Associations with age at onset in the overall group of FTD, FTD/MND, and MND probands.** Three variants are shown that demonstrate a significant association with age at onset in *C9ORF72* expansion carriers (rs7018487:T > G [*UBAP1*; panel **A**], rs6052771:A > G [*PRNP*; panel **B**], and rs7403881:G > C [*MT-Ie*; panel **C**]). In each panel, the mean in the given group is denoted by a solid horizontal line; associations are specified in Table 2 and genotype frequencies in Additional file 1: Table S1.

Our primary analysis focused on the 265 probands carrying *C9ORF72* repeat expansions with FTD, FTD/MND, or MND. Under a false discovery rate (FDR) of 10%, none of the variants studied was significantly associated with disease risk, neither in our overall group nor in any of our disease subgroups. Age at onset analysis, however, revealed three significant associations in our overall group (Table 2; Figure 1). Each additional minor allele of rs7018487 (ubiquitin-associated protein 1 [*UBAP1*]) was associated with a decrease in mean age at onset of 2.62 years (p-value = 0.003; additive genotypic model). For rs6052771 (prion protein [*PRNP*]), the mean age at onset was 4.42 years later in probands with two copies of the minor allele, than in probands with at least one copy of the major allele (p-value = 0.003; recessive genotypic model). Probands carrying at least one copy of the minor allele in rs7403881 (metallothionein 1 E [*MT*-*Ie*] haploblock), demonstrated a delay of 3.95 years in mean age at onset as compared to probands homozygous for the major allele (p-value = 0.003; dominant genotypic model). We did not detect significant associations for any of the disease subgroups.

**Figure 2:**
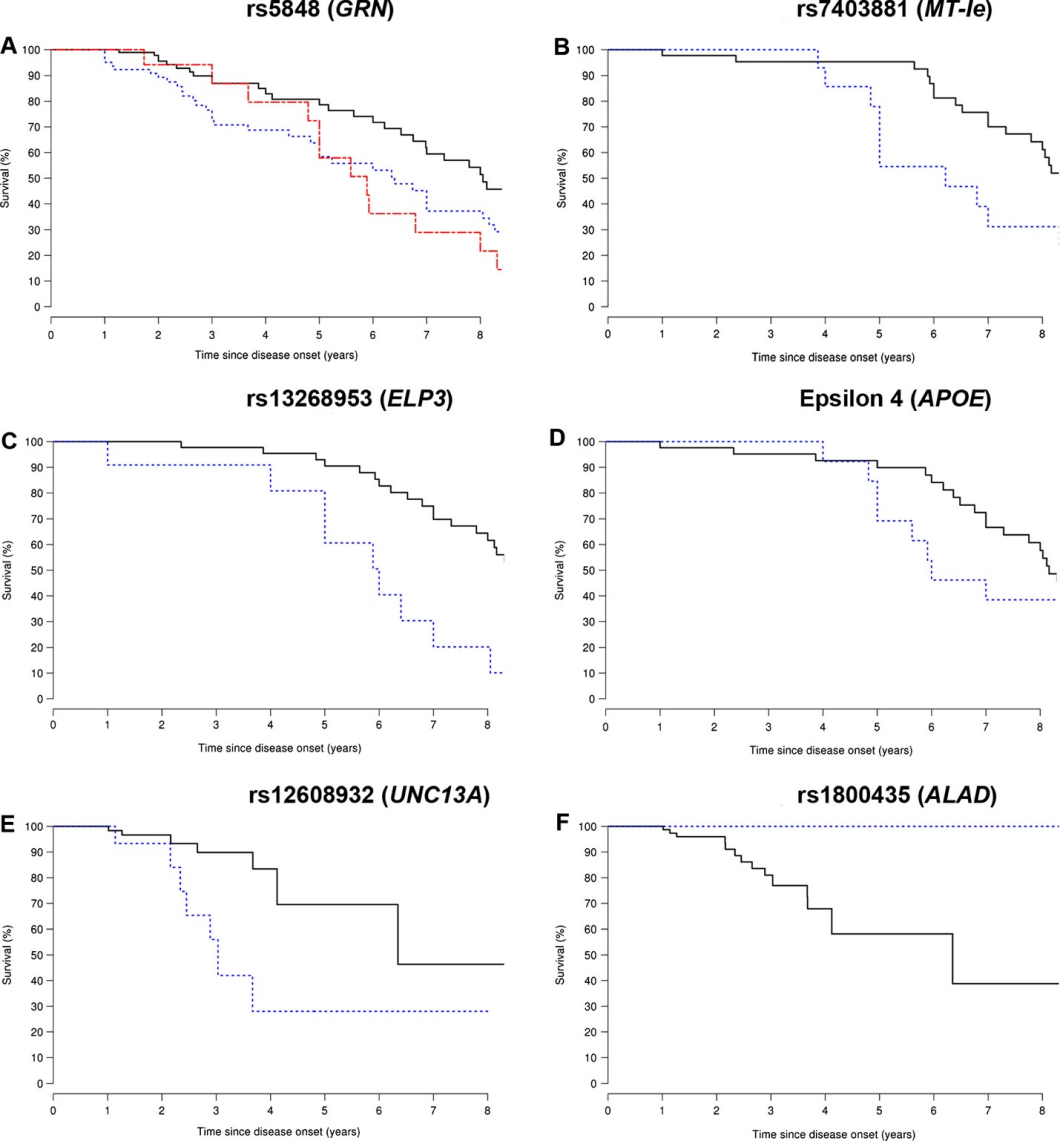
**Variants significantly associated with survival after onset.** Six significant associations with survival after onset are presented (rs5848:G > A [*GRN*; panel **A**], rs7403881:G > C [*MT-Ie*; panel **B**], rs13268953:A > G [*ELP3*; panel **C**], the epsilon 4 allele:E4- > E4+ [*APOE*; panel **D**], rs12608932:A > C [*UNC13A*; panel **E**], and rs1800435G > C [*ALAD*; panel **F**]). When three curves are shown (rs5848), zero copies of the minor allele are displayed in black, one copy of the minor allele is displayed in blue, and two copies of the minor allele are displayed in red. If two curves are present (other variants), then the common genotype is shown in black and the rare genotype is shown in blue.

In the 221 FTD, FTD/MND, and MND probands with information available regarding survival after onset, median follow-up length after onset was three years (range: 4 months – 24 years [FTD: 1 year – 24 years, FTD/MND: 10 months – 24 years, MND: 4 months – 9 years]). The survival after onset analysis resulted in significant associations with six variants (Table 2). Of those associations, one was present in our overall group, three were present in our FTD subgroup, and two were present in our MND subgroup. When concentrating on our overall group (Table 2; Figure 2), we noted a significant association only for rs5848 (granulin precursor [*GRN*]; relative risk [RR] = 1.64; p-value = 0.001; additive genotypic model). However, we also performed an additional analysis to evaluate the combined effect of two other variants, rs13268953 and rs6985069 (elongator acetyltransferase complex subunit 3 [*ELP3*]; not in linkage disequilibrium [LD]), on survival after onset, especially because these variants both showed non-significant trends towards an association and were located near the same gene. When combining these variants, we did detect a significant association with survival after onset (p-value = 0.001; Additional file 1: Table S4).

In our disease subgroups (Table 2; Figure 2), we observed significant associations in our FTD probands for rs7403881 (*MT*-*Ie*; RR = 3.81; p-value = 0.001; recessive genotypic model), rs13268953 (*ELP3*; RR = 3.65; p-value = 0.003; recessive genotypic model), and the epsilon 4 allele (apolipoprotein E [*APOE*]; rs429358 and rs7412; RR = 3.13; p-value = 0.004; dominant genotypic model). In our MND probands, significant associations were found for rs12608932 (unc-13 homolog A, C. elegans [*UNC13A*]; RR = 5.65; p-value = 0.003; recessive genotypic model) and rs1800435 (delta-aminolevulinate dehydratase [*ALAD*]; 0.0% death in carriers of minor allele versus 15.9% in non-carriers; p-value = 0.003; dominant genotypic model).

Of note, all results of statistical analyses involving disease risk, age at onset and survival after onset were very similar when including individuals who were family members or who had received another diagnosis, and also when additionally adjusting models for age in the disease risk analysis (data not shown).

## Discussion

This study was designed to help elucidate the clinical variability observed in *C9ORF72* expansion carriers. We investigated variants previously implicated in FTD and/or MND, and determined their effects in a unique cohort of subjects with known pathogenic expansions in *C9ORF72*. Excitingly, we discovered eight variants that may assist in explaining the reported phenotypic variability, especially with regard to age at onset and survival after onset (Table 2). Although it should be stressed that replication is needed, our results represent a major step forward in the search for genetic modifiers, and they provide directions for future validation and meta-analytical studies.

We identified one single nucleotide polymorphism (SNP) located near *UBAP1* (rs7018487) that was associated with age at onset in our overall group of *C9ORF72* expansion carriers (p-value = 0.003). UBAP1 functions in ubiquitin-dependent sorting at the multivesicular body (MVB), and depletion of UBAP1 severely disrupts this complex process [16, 17]. Variants in *UBAP1* have already been linked to FTD risk, and colocalization of UBAP1 and TDP-43 in neuronal cytoplasmic inclusions has been demonstrated [18]. Our results also revealed an association between *PRNP* (rs6052771; in LD with rs1799990) and age at onset in our overall group (p-value = 0.003). The contribution of *PRNP* to the pathogenesis of FTD and/or MND has not been studied thoroughly [19, 20], and consequently, little is known about its effects on these diseases. One study, however, reported an association of *PRNP* with age at onset in a small number of FTD patients harboring *GRN* mutations [21], supporting the premise of a common underlying mechanism.

Moreover, we discovered a variant in metallothionein (rs7403881) that is associated with a delayed age at onset in our overall group (p-value = 0.003). In addition to this delay, we detected a decrease in survival after onset in our FTD subgroup (p-value = 0.001). Currently, only a few studies investigating FTD and/or MND have focused on the metallothionein family, which is involved in antioxidant defense [22]. One of these studies suggested that rs7403881 increases MND risk [23]. A recent study in superoxide dismutase-1 (*Sod1*) mice, revealed that overexpression of metallothioneins slows disease progression and extends lifespan [24]. Further evidence for a potential role of oxidative stress is provided by the association between survival after onset and a coding SNP in *ALAD* (rs1800435; p-value = 0.003). The ALAD enzyme influences susceptibility to lead exposure, which may contribute to MND risk; although studies published thus far are insufficient for a definitive conclusion [25–27].

Interestingly, we also found a significant association between a functional SNP in *GRN* (rs5848; 3’-untranslated region [UTR]) and survival after onset in our overall group. It has already been reported that carriers homozygous for the minor allele of rs5848 demonstrate an increased FTD risk as compared to homozygous major allele carriers [28], but no associations with either FTD risk or age at onset were observed in other studies [21, 29–32]. Thus, although the contribution of *GRN* SNPs to neurodegenerative diseases has not been elucidated, our present finding suggests that *GRN* is associated with survival after onset in carriers of *C9ORF72* repeat expansions (p-value = 0.001).

We also examined two variants near *ELP3* (rs13268953 and rs6985069; not in LD). *ELP3* is a component of the RNA polymerase II complex, and as such, is involved in the acetylation of histones H3 and H4 to make DNA accessible for transcription [33, 34]. Importantly, another type of histone modification has already been implicated in *C9ORF72* expansion carriers: a recent report demonstrated that trimethylation of lysine residues within histones H3 and H4 might reduce *C9ORF72* expression in expansion carriers [35]. An association study and mutagenesis screen have also exposed associations between *ELP3* and MND susceptibility [36], representing one of many FTD and/or MND-associated genes that function in RNA-processing pathways [37]. Our present findings are in agreement with these studies, as shown by the combined effects of these *ELP3* SNPs in our overall group (p-value = 0.001), and one *ELP3* SNP (rs13268953) in our FTD subgroup (p-value = 0.003).

In addition, we assessed *APOE*, a gene that has been carefully investigated, particularly in patients with dementia. A recent meta-analysis included 28 case–control studies, and demonstrated that the epsilon 4 allele increases susceptibility to FTD [38]. Interestingly, we discovered that the *APOE* epsilon 4 allele was associated with a decline in survival after onset in our FTD subgroup (p-value = 0.004).

Our last potential modifier (rs12608932), an intronic SNP in *UNC13A*, has been identified through a genome-wide association study in MND patients [39]. This finding was strengthened by an analysis of expression quantitative trait loci (eQTLs) that demonstrated genome-wide significance for *UNC13A* [40]. UNC13A is involved in neurotransmitter release [41], a tightly regulated process that is thought to be disrupted in MND patients. Our results show that variants in *UNC13A* are also associated with survival after onset in the presence of a *C9ORF72* repeat expansion: we detected an association in our MND subgroup (p-value = 0.003).

We would like to reiterate that we performed a systematic study of variants reported in the literature. For many variants, however, previous findings were inconclusive and based on our current discoveries we speculate that some of the seemingly conflicting results are due to differences in the composition (and size) of study cohorts, most importantly: (1) the number of patients with predominant FTD, predominant MND or a mixture of both diseases, (2) the percentage of subjects with a pathologically confirmed diagnosis, and (3) the subset of individuals with pathogenic mutations in particular FTD and/or MND-associated genes (such as *C9ORF72*). Hence, because of our present findings and results of aforementioned studies, reinvestigation of previously published data after exclusion of certain subgroups seems warranted, and new well-sized studies should be performed concentrating on these subgroups, in order to determine the specificity of results.

In our study, we used an FDR rather than a family-wise error rate (FWER)-controlling procedure for multiple testing adjustments. The FDR procedure is relatively new, and controlling the FDR is a valid method to adjust for multiple comparisons [42]. An FDR correction, however, is less conservative than an FWER correction and its interpretation is different (Methods). We used an FDR of 10%, which means that for each group of statistically significant associations we would expect the vast majority (90%) to be real (i.e. for each group only 1 out of 10 significant findings is expected to be false). Naturally, there is always a balance between the two different types of statistical error that can occur for any given conclusion – a type I error (i.e. a false-positive association) and a type II error (i.e. a false-negative association), both of which are undesirable. Because the balance tips more in the direction of type I error for the FDR than for the FWER procedures, it is important to highlight that our results, though promising, do require validation.

Additionally, it should be noted that we focused our article on those associations that remained significant after adjustment for multiple testing. Future studies could investigate nominally significant associations (Additional file 1: Table S2 and Additional file 1: Table S3) in larger cohorts and/or meta-analyses, to determine whether any of these potential associations contribute to the pleiotropy detected in *C9ORF72* expansion carriers. Other studies could also concentrate on variants not included in our present study (i.e. recently published variants); especially since it seems plausible that more variants (either known or unknown) modify the phenotype of *C9ORF72* expansion carriers. Furthermore, our study was designed to investigate associations with disease risk (i.e. by comparing patients and controls) and to identify factors that could modify age at onset or survival after onset. Interestingly, some of the associations we observed were only significant in the phenotypic subgroup for which the risk variant was originally reported; for example, *APOE* genotypes only affected survival after onset in our subgroup of *C9ORF72* expansion carriers with FTD, whereas the *UNC13A* variant only affected survival after onset in our MND subgroup. To further investigate the clinical phenotype, a larger number of expansion carriers with either FTD or MND is needed (e.g. international genome-wide association study), so that direct comparisons of expansion carriers with FTD and MND could be performed.

## Conclusions

Our present study reveals eight variants that may account for the phenotypic variability reported in *C9ORF72* expansion carriers. These variants strongly emphasize the importance of proper protein degradation, antioxidant defense, and processing of RNA. Although identified genes (and their corresponding pathways) have already been linked to FTD and/or MND, it was unclear whether they were able to act as disease modifiers on the background of a *C9ORF72* repeat expansion. Our findings, thus, underscore the complex interplay between many factors that influence the occurrence and prognosis of these destructive diseases, particularly in *C9ORF72* expansion carriers. Though large for a study of *C9ORF72* expansion carriers, our findings result from a relatively small sample size, and therefore, repeated replication and meta-analyses will be necessary to increase our understanding of these potential genetic disease modifiers. With that said, the factors identified in this study may represent excellent targets for novel treatments, including preventative treatment strategies, and for the development of predictive tests aiming at the continuum of FTD and MND.

## Methods

### Subjects

We collected DNA from a cohort of 330 *C9ORF72* expansion carriers, obtained at the Mayo Clinic (n = 121), Coriell Research Institute (n = 71), University of British Columbia, Canada (n = 58), University of California, San Francisco (n = 38), Robarts Research Institute (n = 11), Northwestern University Feinberg School of Medicine (n = 9), Drexel University College of Medicine (n = 7), University of Western Ontario, Canada (n = 7), Banner Sun Health Research Institute (n = 5), and University of Tübingen (n = 3). Based on available clinical and/or pathological data, these subjects were diagnosed with FTD (n = 91), FTD/MND (n = 78) or MND (n = 127), with another diagnosis (n = 7; e.g. dementia due to Alzheimer’s disease, alcohol abuse or behavioral impairment), or they were asymptomatic at time of last evaluation (n = 27; mean age at evaluation: 43.6 ± 12.7 standard deviation [SD]). Of those expansion carriers 45.2% (n = 149) were female, their mean age was 59.4 ± 10.0 years, their main age at onset was 56.5 ± 9.1 years, and 37.3% (n = 123) had received a neuropathological diagnosis (Table 1). Age at onset was estimated based on the appearance of the first disease symptoms, namely progressive cognitive dysfunction in judgment, language, or memory; or changes in behavior or personality (FTD patients); or fasciculations, muscle weakness, falls, dysarthria, and dysphagia (MND patients). When symptoms of both FTD and MND were noted, the earliest observation of decline was recorded for age at onset. Survival after onset was defined as the interval between age at onset of disease symptoms and the age at death for deceased patients, and as the interval between age at onset and present age for other patients (when follow-up data was available).

We also included neurologically normal controls (n = 374), of whom 46.0% (n = 172) were female, and whose mean age was 61.2 ± 10.2 years. All subjects agreed to be in the study, and biological samples were obtained after informed consent with ethical committee approval from the respective institutions. Approval for the genetic analyses was performed in agreement with ethical committee approval at Mayo Clinic

### Genotyping

*C9ORF72* expansion carriers were identified using our previously published 2-step PCR protocol [7]; Southern blotting techniques were employed to confirm the presence of the repeat expansion when sufficient high quality DNA was available (>25% of expansion carriers) [14]. To select candidates that could potentially act as disease modifiers in carriers of *C9ORF72* repeat expansions, we performed a literature search on PubMed (August 2012) that revealed all publications on a combination of FTD and/or MND with SNPs. Subsequently, we selected one or two variants per gene possibly associated with these diseases (top SNPs were preferred), which were suitable for the Sequenom MassArray iPLEX platform (San Diego, CA, USA) and could be incorporated in Sequenom panels; these variants were analyzed with Typer 4.0 software. Sequenom genotype data was supplemented with five Taqman SNP genotyping assays (C_3084793_20, C_1085600_10, C_2070266_20, C_8921964_20, and C_7563736_10; Invitrogen, Carlsbad, CA, USA) performed on a 7900HT Fast Real Time PCR system; genotype calls were made using SDS 2.4 software (Applied Biosystems, Foster City, CA, USA). After genotyping, we excluded SNPs with a significant deviation from the Hardy-Weinberg equilibrium (HWE) in our control cohort (rs45559331 and rs6903982), rare SNPs with a minor allele frequency (MAF) of less than 1% in expansion carriers and controls (rs121909536, rs75654767, rs121909541, rs140547520, rs80265967, rs80356715, and rs35070491), and SNPs with a call rate below 95% (rs4680, rs4859146, rs854560, rs7277748, rs4880, and rs2275294). In total, 36 variants were included in our analysis (Additional file 1: Table S1); the call rate of these variants was greater than 99% and none of these variants was in LD. All genetic analyses were performed at the Mayo Clinic, and genotypes were assigned using all of the data from the study simultaneously.

### Statistical analysis

In order to satisfy the statistical assumption of independent measurements, our primary analysis focused on a subset of *C9ORF72* expansion carriers: 265 unrelated probands with FTD (n = 74), FTD/MND (n = 71), or MND (n = 120). We performed secondary analyses, however, that included the remaining expansion carriers to examine the sensitivity of our results. The entire cohort of *C9ORF72* expansion carriers was assessed, and also disease subgroups separately (FTD, FTD/MND and MND). First, we used logistic regression models adjusted for gender to evaluate associations of each of the 36 variants with disease risk; odds ratios (ORs) and 95% confidence intervals (CIs) were estimated. In addition, we examined associations of each of these variants with age at onset using linear regression models adjusted for gender and disease subgroup; while associations with survival after onset were assessed using Cox proportional hazards regression models adjusted for age at onset, gender, and disease subgroup. Regression coefficients (interpreted as changes in mean age at onset) and 95% CIs were estimated in the age at onset linear regression analysis; whereas in Cox regression analysis, RRs and 95% CIs were estimated, and data was censored at last follow-up. Each variant was investigated under an additive genotypic model (effect of each additional minor allele), a dominant genotypic model (presence versus absence of the minor allele), and a recessive genotypic model (presence versus absence of two copies of the minor allele). Models were not adjusted for *C9ORF72* expansion size, since expansion sizes were only available for a subset of samples (>25%) and they were estimated in DNA obtained from various tissues, which hampers analyses [14]. In order to reduce the chance of spurious findings and non-informative tests, association analyses were not performed for variants with fewer than ten carriers of the minor allele in the given group, or under an additive or recessive genotypic model when fewer than ten rare homozygotes were present in the given group.

To account for multiple testing, we made an adjustment separately for each disease group and separately for each outcome measure (disease risk, age at onset, and survival after onset). Given the relatively small sample size of this study, controlling the FWER (i.e. the probability of any false-positive finding among the entire group of tests) at 5% using a procedure such as a single-step minP permutation correction [43] would result in very low power to detect associations. We, therefore, opted for an alternative approach and utilized an FDR correction [44]. This increasingly used method has a different interpretation than FWER-controlling procedures; an FDR procedure attempts to control the expected proportion of false-positive findings among those associations considered significant. Note that due to this difference in interpretation, the FDR does not necessarily need to be controlled at 5%, only at a reasonable level to allow for high confidence in results, which was deemed at 10% for our study [42]. All statistical tests were two-sided, and were performed using SAS (version 9.2; SAS Institute, Inc., Cary, NC, USA) and R Statistical Software (version 2.14.0; R Foundation for Statistical Computing, Vienna, Austria).

## Electronic supplementary material

Additional file 1: **Genotype counts and frequencies (Table S1), Associations with age at onset under additive, dominant and recessive models in the overall group of FTD, FTD/MND, and MND probands (n = 243; Table S2), Associations with survival after onset under additive, dominant and recessive models in the overall group of FTD, FTD/MND, and MND probands (n = 221; Table S3a), Associations with survival after onset under additive, dominant and recessive models in FTD probands (n = 58; Table S3b), Associations with survival after onset under additive, dominant and recessive models in MND probands (n = 107; Table S3c), Combinations of** ***ELP3*** **variants rs13268953 and rs6985069 in relation to survival after onset in the overall group (Table S4).** (DOCX 89 KB)

## Abbreviations

FTD: Frontotemporal dementia
MND: Motor neuron disease
TDP-43: transactive response DNA-binding protein 43
*C9ORF72*: Chromosome 9 open reading frame 72
*TMEM106B*: Transmembrane protein 106 B
*ATXN2*: Ataxin-2
FDR: False discovery rate
*UBAP1*: Ubiquitin-associated protein 1
*PRNP*: Prion protein
*MT*-*Ie*: Metallothionein 1 E
*GRN*: Granulin precursor
RR: Relative risk
*ELP3*: Elongator acetyltransferase complex subunit 3
LD: Linkage disequilibrium
*APOE*: Apolipoprotein E
*UNC13A*: unc-13 homolog A, C. elegans
*ALAD*: Delta-aminolevulinate dehydratase
SNP: Single nucleotide polymorphism
MVB: Multivesicular body
*Sod1*: Superoxide dismutase-1
UTR: Untranslated region
eQTLs: Expression quantitative trait loci
FWER: Family-wise error rate
SD: Standard deviation
HWE: Hardy-Weinberg equilibrium
MAF: Minor allele frequency
OR: Odds ratio
CI: Confidence interval.
