# Supplementary material for: Genetic modifiers in carriers of repeat expansions in the C9ORF72 gene
Source: Mol Neurodegener. 2014 Sep 20;9:38. doi: 10.1186/1750-1326-9-38 (PMC4190282; doi:10.1186/1750-1326-9-38)
Supplement: Supplementary file 1 — Additional file 1: Genotype counts and frequencies (Table S1), Associations with age at onset under additive, dominant and recessive models in the overall group of FTD, FTD/MND, and MND probands (n = 243; Table S2), Associations with survival after onset under additive, dominant and recessive models in the overall group of FTD, FTD/MND, and MND probands (n = 221; Table S3a), Associations with survival after onset under additive, dominant and recessive models in FTD probands (n = 58; Table S3b), Associations with survival after onset under additive, dominant and recessive models in MND probands (n = 107; Table S3c), Combinations of ELP3 variants rs13268953 and rs6985069 in relation to survival after onset in the overall group (Table S4). (DOCX 89 KB) [file 13024_2014_548_MOESM1_ESM.docx]

**Additional File**

**Genetic modifiers in carriers of repeat expansions in the *C9ORF72* gene**

Marka van Blitterswijk, Bianca Mullen, Aleksandra Wojtas, Michael G Heckman, Nancy N Diehl*,* Matthew C Baker, Mariely DeJesus-Hernandez, Patricia H Brown, Melissa E Murray, Ging-Yuek R Hsiung, Heather Stewart, Anna M Karydas, Elizabeth Finger, Andrew Kertesz, Eileen H Bigio, Sandra Weintraub, Marsel Mesulam, Kimmo J Hatanpaa, Charles L White III, Manuela Neumann, Michael J Strong, Thomas G Beach, Zbigniew K Wszolek, Carol Lippa, Richard Caselli, Leonard Petrucelli, Keith A Josephs, Joseph E Parisi, David S Knopman, Ronald C Petersen, Ian R Mackenzie, William W Seeley, Lea T Grinberg, Bruce L Miller, Kevin B Boylan, Neill R Graff-Radford, Bradley F Boeve, Dennis W Dickson, and Rosa Rademakers

**Genotype counts and frequencies (Table S1), Associations with age at onset under additive, dominant and recessive models in the overall group of FTD, FTD/MND, and MND probands (n = 243; Table S2), Associations with survival after onset under additive, dominant and recessive models in the overall group of FTD, FTD/MND, and MND probands (n = 221; Table S3a), Associations with survival after onset under additive, dominant and recessive models in FTD probands (n = 58; Table S3b), Associations with survival after onset under additive, dominant and recessive models in MND probands (n = 107; Table S3c), Combinations of *ELP3* variants rs13268953 and rs6985069 in relation to survival after onset in the overall group (Table S4)**

**Table S1 Genotype counts and frequencies**

| Variant (gene) | Minor allele count and frequency | Major allele count and frequency | Genotype 1 count and frequency | Genotype 2 count and frequency | Genotype 3 count and frequency |
| --- | --- | --- | --- | --- | --- |
| rs10260404 (*DPP6*) |  |  |  |  |  |
| All Cases | C: 229 (34.8%) | T: 429 (65.2%) | TT: 143 (43.5%) | TC: 143 (43.5%) | CC: 43 (13.1%) |
| Proband cases | C: 190 (36.0%) | T: 338 (64.0%) | TT: 112 (42.4%) | TC: 114 (43.2%) | CC: 38 (14.4%) |
| FTD | C: 49 (33.6%) | T: 97 (66.4%) | TT: 33 (45.2%) | TC: 31 (42.5%) | CC: 9 (12.3%) |
| FTD/MND | C: 51 (35.9%) | T: 91 (64.1%) | TT: 32 (45.1%) | TC: 27 (38.0%) | CC: 12 (16.9%) |
| MND | C: 90 (37.5%) | T: 150 (62.5%) | TT: 47 (39.2%) | TC: 56 (46.7%) | CC: 17 (14.2%) |
| Controls | C: 306 (40.9%) | T: 442 (59.1%) | TT: 131 (35%) | TC: 180 (48.1%) | CC: 63 (16.8%) |
| rs10487132 (*PON3*) |  |  |  |  |  |
| All Cases | G: 261 (39.5%) | A: 399 (60.5%) | AA: 130 (39.4%) | GA: 139 (42.1%) | GG: 61 (18.5%) |
| Proband cases | G: 209 (39.4%) | A: 321 (60.6%) | AA: 103 (38.9%) | GA: 115 (43.4%) | GG: 47 (17.7%) |
| FTD | G: 64 (43.2%) | A: 84 (56.8%) | AA: 25 (33.8%) | GA: 34 (45.9%) | GG: 15 (20.3%) |
| FTD/MND | G: 44 (31.0%) | A: 98 (69.0%) | AA: 35 (49.3%) | GA: 28 (39.4%) | GG: 8 (11.3%) |
| MND | G: 101 (42.1%) | A: 139 (57.9%) | AA: 43 (35.8%) | GA: 53 (44.2%) | GG: 24 (20.0%) |
| Controls | G: 299 (40.1%) | A: 447 (59.9%) | AA: 136 (36.5%) | GA: 175 (46.9%) | GG: 62 (16.6%) |
| rs1052352 (*FUS*) |  |  |  |  |  |
| All Cases | C: 298 (45.3%) | T: 360 (54.7%) | TT: 100 (30.4%) | TC: 160 (48.6%) | CC: 69 (21.0%) |
| Proband cases | C: 241 (45.5%) | T: 289 (54.5%) | TT: 82 (30.9%) | TC: 125 (47.2%) | CC: 58 (21.9%) |
| FTD | C: 61 (41.2%) | T: 87 (58.8%) | TT: 27 (36.5%) | TC: 33 (44.6%) | CC: 14 (18.9%) |
| FTD/MND | C: 66 (46.5%) | T: 76 (53.5%) | TT: 21 (29.6%) | TC: 34 (47.9%) | CC: 16 (22.5%) |
| MND | C: 114 (47.5%) | T: 126 (52.5%) | TT: 34 (28.3%) | TC: 58 (48.3%) | CC: 28 (23.3%) |
| Controls | C: 350 (46.8%) | T: 398 (53.2%) | TT: 109 (29.1%) | TC: 180 (48.1%) | CC: 85 (22.7%) |
| rs10971977 (*UBAP1*) |  |  |  |  |  |
| All Cases | T: 303 (45.9%) | A: 357 (54.1%) | AA: 97 (29.4%) | AT: 163 (49.4%) | TT: 70 (21.2%) |
| Proband cases | T: 252 (47.5%) | A: 278 (52.5%) | AA: 73 (27.5%) | AT: 132 (49.8%) | TT: 60 (22.6%) |
| FTD | T: 67 (45.3%) | A: 81 (54.7%) | AA: 23 (31.1%) | AT: 35 (47.3%) | TT: 16 (21.6%) |
| FTD/MND | T: 72 (50.7%) | A: 70 (49.3%) | AA: 19 (26.8%) | AT: 32 (45.1%) | TT: 20 (28.2%) |
| MND | T: 113 (47.1%) | A: 127 (52.9%) | AA: 31 (25.8%) | AT: 65 (54.2%) | TT: 24 (20.0%) |
| Controls | T: 316 (42.4%) | A: 430 (57.6%) | AA: 116 (31.1%) | AT: 198 (53.1%) | TT: 59 (15.8%) |
| rs1130409 (*APEX1*) |  |  |  |  |  |
| All Cases | G: 291 (44.4%) | T: 365 (55.6%) | TT: 99 (30.2%) | GT: 167 (50.9%) | GG: 62 (18.9%) |
| Proband cases | G: 234 (44.5%) | T: 292 (55.5%) | TT: 79 (30.0%) | GT: 134 (51.0%) | GG: 50 (19.0%) |
| FTD | G: 67 (45.3%) | T: 81 (54.7%) | TT: 21 (28.4%) | GT: 39 (52.7%) | GG: 14 (18.9%) |
| FTD/MND | G: 62 (44.3%) | T: 78 (55.7%) | TT: 23 (32.9%) | GT: 32 (45.7%) | GG: 15 (21.4%) |
| MND | G: 105 (44.1%) | T: 133 (55.9%) | TT: 35 (29.4%) | GT: 63 (52.9%) | GG: 21 (17.6%) |
| Controls | G: 349 (46.7%) | T: 399 (53.3%) | TT: 100 (26.7%) | GT: 199 (53.2%) | GG: 75 (20.1%) |
| rs11701 (*ANG*) |  |  |  |  |  |
| All Cases | G: 70 (10.7%) | T: 574 (87.8%) | TT: 249 (76.1%) | TG: 76 (23.2%) | GG: 2 (0.6%) |
| Proband cases | G: 70 (13.3%) | T: 456 (86.7%) | TT: 195 (74.1%) | TG: 66 (25.1%) | GG: 2 (0.8%) |
| FTD | G: 21 (14.4%) | T: 125 (85.6%) | TT: 53 (72.6%) | TG: 19 (26%) | GG: 1 (1.4%) |
| FTD/MND | G: 18 (12.7%) | T: 124 (87.3%) | TT: 53 (74.6%) | TG: 18 (25.4%) | GG: 0 (0%) |
| MND | G: 31 (13%) | T: 207 (87%) | TT: 89 (74.8%) | TG: 29 (24.4%) | GG: 1 (0.8%) |
| Controls | G: 91 (12.2%) | T: 655 (87.8%) | TT: 286 (76.7%) | TG: 83 (22.3%) | GG: 4 (1.1%) |
| rs12608932 (*UNC13A*) |  |  |  |  |  |
| All Cases | C: 258 (39.2%) | A: 400 (60.8%) | AA: 131 (39.8%) | CA: 138 (41.9%) | CC: 60 (18.2%) |
| Proband cases | C: 199 (37.7%) | A: 329 (62.3%) | AA: 113 (42.8%) | CA: 103 (39.0%) | CC: 48 (18.2%) |
| FTD | C: 51 (34.5%) | A: 97 (65.5%) | AA: 33 (44.6%) | CA: 31 (41.9%) | CC: 10 (13.5%) |
| FTD/MND | C: 55 (38.7%) | A: 87 (61.3%) | AA: 30 (42.3%) | CA: 27 (38.0%) | CC: 14 (19.7%) |
| MND | C: 93 (39.1%) | A: 145 (60.9%) | AA: 50 (42.0%) | CA: 45 (37.8%) | CC: 24 (20.2%) |
| Controls | C: 274 (36.6%) | A: 474 (63.4%) | AA: 149 (39.8%) | CA: 176 (47.1%) | CC: 49 (13.1%) |
| rs13268953 (*ELP3*) |  |  |  |  |  |
| All Cases | G: 282 (42.7%) | A: 378 (57.3%) | AA: 108 (32.7%) | GA: 162 (49.1%) | GG: 60 (18.2%) |
| Proband cases | G: 231 (43.6%) | A: 299 (56.4%) | AA: 85 (32.1%) | GA: 129 (48.7%) | GG: 51 (19.2%) |
| FTD | G: 67 (45.3%) | A: 81 (54.7%) | AA: 21 (28.4%) | GA: 39 (52.7%) | GG: 14 (18.9%) |
| FTD/MND | G: 64 (45.1%) | A: 78 (54.9%) | AA: 23 (32.4%) | GA: 32 (45.1%) | GG: 16 (22.5%) |
| MND | G: 100 (41.7%) | A: 140 (58.3%) | AA: 41 (34.2%) | GA: 58 (48.3%) | GG: 21 (17.5%) |
| Controls | G: 306 (40.9%) | A: 442 (59.1%) | AA: 133 (35.6%) | GA: 176 (47.1%) | GG: 65 (17.4%) |
| rs1541160 (*KIFAP3*) |  |  |  |  |  |
| All Cases | G: 181 (27.4%) | A: 479 (72.6%) | AA: 171 (51.8%) | GA: 137 (41.5%) | GG: 22 (6.7%) |
| Proband cases | G: 145 (27.4%) | A: 385 (72.6%) | AA: 139 (52.5%) | GA: 107 (40.4%) | GG: 19 (7.2%) |
| FTD | G: 33 (22.3%) | A: 115 (77.7%) | AA: 44 (59.5%) | GA: 27 (36.5%) | GG: 3 (4.1%) |
| FTD/MND | G: 35 (24.6%) | A: 107 (75.4%) | AA: 40 (56.3%) | GA: 27 (38.0%) | GG: 4 (5.6%) |
| MND | G: 77 (32.1%) | A: 163 (67.9%) | AA: 55 (45.8%) | GA: 53 (44.2%) | GG: 12 (10.0%) |
| Controls | G: 219 (29.3%) | A: 529 (70.7%) | AA: 180 (48.1%) | GA: 169 (45.2%) | GG: 25 (6.7%) |
| rs165932 (*PS-1*) |  |  |  |  |  |
| All Cases | C: 289 (43.9%) | A: 369 (56.1%) | AA: 97 (29.5%) | CA: 175 (53.2%) | CC: 57 (17.3%) |
| Proband cases | C: 231 (43.8%) | A: 297 (56.3%) | AA: 80 (30.3%) | CA: 137 (51.9%) | CC: 47 (17.8%) |
| FTD | C: 67 (45.3%) | A: 81 (54.7%) | AA: 22 (29.7%) | CA: 37 (50.0%) | CC: 15 (20.3%) |
| FTD/MND | C: 51 (36.4%) | A: 89 (63.6%) | AA: 26 (37.1%) | CA: 37 (52.9%) | CC: 7 (10.0%) |
| MND | C: 113 (47.1%) | A: 127 (52.9%) | AA: 32 (26.7%) | CA: 63 (52.5%) | CC: 25 (20.8%) |
| Controls | C: 309 (41.3%) | A: 439 (58.7%) | AA: 132 (35.3%) | CA: 175 (46.8%) | CC: 67 (17.9%) |
| rs17350674 (*KIF24*) |  |  |  |  |  |
| All Cases | A: 119 (18.0%) | C: 541 (82.0%) | CC: 221 (67.0%) | CA: 99 (30.0%) | AA: 10 (3%) |
| Proband cases | A: 103 (19.4%) | C: 427 (80.6%) | CC: 171 (64.5%) | CA: 85 (32.1%) | AA: 9 (3.4%) |
| FTD | A: 28 (18.9%) | C: 120 (81.1%) | CC: 49 (66.2%) | CA: 22 (29.7%) | AA: 3 (4.1%) |
| FTD/MND | A: 24 (16.9%) | C: 118 (83.1%) | CC: 47 (66.2%) | CA: 24 (33.8%) | AA: 0 (0.0%) |
| MND | A: 51 (21.3%) | C: 189 (78.8%) | CC: 75 (62.5%) | CA: 39 (32.5%) | AA: 6 (5.0%) |
| Controls | A: 147 (19.7%) | C: 601 (80.3%) | CC: 233 (62.3%) | CA: 135 (36.1%) | AA: 6 (1.6%) |
| rs1799945 (*HFE*) |  |  |  |  |  |
| All Cases | G: 78 (11.9%) | C: 68 (88.1%) | CC: 256 (77.8%) | CG: 68 (20.7%) | GG: 5 (1.5%) |
| Proband cases | G: 60 (11.4%) | C: 468 (88.6%) | CC: 206 (96.7%) | CG: 56 (21.2%) | GG: 2 (0.8%) |
| FTD | G: 16 (11%) | C: 130 (89%) | CC: 57 (78.1%) | CG: 16 (21.9%) | GG: 0 (0%) |
| FTD/MND | G: 17 (12%) | C: 125 (88%) | CC: 54 (76.1%) | CG: 17 (23.9%) | GG: 0 (0%) |
| MND | G: 27 (11.3%) | C: 213 (88.8%) | CC: 95 (79.2%) | CG: 23 (19.2%) | GG: 2 (1.7%) |
| Controls | G: 123 (16.4%) | C: 625 (83.6%) | CC: 263 (70.3%) | CG: 99 (26.5%) | GG: 12 (3.2%) |
| rs1799983 (*NOS3*) |  |  |  |  |  |
| All Cases | T: 231 (35.0%) | G: 429 (65.0%) | GG: 130 (39.4%) | GT: 169 (51.2%) | TT: 31 (9.4%) |
| Proband cases | T: 179 (33.8%) | G: 351 (66.2%) | GG: 110 (41.5%) | GT: 131 (49.4%) | TT: 24 (9.1%) |
| FTD | T: 43 (29.1%) | G: 105 (70.9%) | GG: 35 (47.3%) | GT: 35 (47.3%) | TT: 4 (5.4%) |
| FTD/MND | T: 49 (34.5%) | G: 93 (65.5%) | GG: 31 (43.7%) | GT: 31 (43.7%) | TT: 9 (12.7%) |
| MND | T: 87 (36.3%) | G: 153 (63.8%) | GG: 44 (36.7%) | GT: 65 (54.2%) | TT: 11 (9.2%) |
| Controls | T: 250 (33.4%) | G: 498 (66.6%) | GG: 176 (47.1%) | GT: 146 (39%) | TT: 52 (13.9%) |
| rs1800435 (*ALAD*) |  |  |  |  |  |
| All Cases | C: 47 (7.1%) | G: 613 (92.9%) | GG: 286 (86.7%) | GC: 41 (12.4%) | CC: 3 (0.9%) |
| Proband cases | C: 42 (7.9%) | G: 488 (92.1%) | GG: 226 (85.3%) | GC: 36 (13.6%) | CC: 3 (1.1%) |
| FTD | C: 7 (4.7%) | G: 141 (95.3%) | GG: 68 (91.9%) | GC: 5 (6.8%) | CC: 1 (1.4%) |
| FTD/MND | C: 9 (6.3%) | G: 133 (93.7%) | GG: 62 (87.3%) | GC: 9 (12.7%) | CC: 0 (0.0%) |
| MND | C: 26 (10.8%) | G: 214 (89.2%) | GG: 96 (80.0%) | GC: 22 (18.3%) | CC: 2 (1.7%) |
| Controls | C: 56 (7.5%) | G: 688 (92.5%) | GG: 319 (85.8%) | GC: 50 (13.4%) | CC: 3 (0.8%) |
| rs1801133 (*MTHFR*) |  |  |  |  |  |
| All Cases | T: 233 (35.3%) | C: 427 (64.7%) | CC: 143 (43.3%) | TC: 141 (42.7%) | TT: 46 (13.9%) |
| Proband cases | T: 191 (36.0%) | C: 339 (64.0%) | CC: 113 (42.6%) | TC: 113 (42.6%) | TT: 39 (14.7%) |
| FTD | T: 48 (32.4%) | C: 100 (67.6%) | CC: 35 (47.3%) | TC: 30 (40.5%) | TT: 9 (12.2%) |
| FTD/MND | T: 51 (35.9%) | C: 91 (64.1%) | CC: 30 (42.3%) | TC: 31 (43.7%) | TT: 10 (14.1%) |
| MND | T: 92 (38.3%) | C: 148 (61.7%) | CC: 48 (40.0%) | TC: 52 (43.3%) | TT: 20 (16.7%) |
| Controls | T: 232 (31.0%) | C: 516 (69.0%) | CC: 179 (47.9%) | TC: 158 (42.2%) | TT: 37 (9.9%) |
| rs2010963 (*VEGF*) |  |  |  |  |  |
| All Cases | C: 201 (30.5%) | G: 459 (69.5%) | GG: 162 (49.1%) | GC: 135 (40.9%) | CC: 33 (10.0%) |
| Proband cases | C: 171 (32.3%) | G: 359 (67.7%) | GG: 125 (47.2%) | GC: 109 (41.1%) | CC: 31 (11.7%) |
| FTD | C: 54 (36.5%) | G: 94 (63.5%) | GG: 31 (41.9%) | GC: 32 (43.2%) | CC: 11 (14.9%) |
| FTD/MND | C: 37 (26.1%) | G: 105 (73.9%) | GG: 40 (56.3%) | GC: 25 (35.2%) | CC: 6 (8.5%) |
| MND | C: 80 (33.3%) | G: 160 (66.7%) | GG: 54 (45.0%) | GC: 52 (43.3%) | CC: 14 (11.7%) |
| Controls | C: 227 (30.5%) | G: 517 (69.5%) | GG: 186 (50%) | GC: 145 (39.0%) | CC: 41 (11.0%) |
| rs2279720 (*CHMP2B*) |  |  |  |  |  |
| All Cases | A: 48 (7.3%) | G: 612 (92.7%) | GG: 282 (85.5%) | GA: 48 (14.5%) | AA: 0 (0.0%) |
| Proband cases | A: 35 (6.6%) | G: 495 (93.4%) | GG: 230 (86.8%) | GA: 35 (13.2%) | AA: 0 (0.0%) |
| FTD | A: 9 (6.1%) | G: 139 (93.9%) | GG: 65 (87.8%) | GA: 9 (12.2%) | AA: 0 (0.0%) |
| FTD/MND | A: 7 (4.9%) | G: 135 (95.1%) | GG: 64 (90.1%) | GA: 7 (9.9%) | AA: 0 (0.0%) |
| MND | A: 19 (7.9%) | G: 221 (92.1%) | GG: 101 (84.2%) | GA: 19 (15.8%) | AA: 0 (0.0%) |
| Controls | A: 54 (7.2%) | G: 694 (92.8%) | GG: 323 (86.4%) | GA: 48 (12.8%) | AA: 3 (0.8%) |
| rs2306677 (*ITPR2*) |  |  |  |  |  |
| All Cases | T: 51 (7.9%) | C: 597 (92.1%) | CC: 274 (84.6%) | CT: 49 (15.1%) | TT: 1 (0.3%) |
| Proband cases | T: 44 (8.5%) | C: 474 (91.5%) | CC: 216 (83.4%) | CT: 42 (16.2%) | TT: 1 (0.4%) |
| FTD | T: 16 (11.0%) | C: 130 (89.0%) | CC: 57 (78.1%) | CT: 16 (21.9%) | TT: 0 (0.0%) |
| FTD/MND | T: 5 (3.6%) | C: 135 (96.4%) | CC: 65 (92.9%) | CT: 5 (7.1%) | TT: 0 (0.0%) |
| MND | T: 23 (9.9%) | C: 209 (90.1%) | CC: 94 (81.0%) | CT: 21 (18.1%) | TT: 1 (0.9%) |
| Controls | T: 61 (8.2%) | C: 685 (91.8%) | CC: 313 (83.9%) | CT: 59 (15.8%) | TT: 1 (0.3%) |
| rs407135 (*SLC11A2*) |  |  |  |  |  |
| All Cases | C: 176 (26.7%) | A: 484 (73.3%) | AA: 177 (53.6%) | CA: 130 (39.4%) | CC: 23 (7.0%) |
| Proband cases | C: 137 (25.8%) | A: 393 (74.2%) | AA: 147 (55.5%) | CA: 99 (37.4%) | CC: 19 (7.2%) |
| FTD | C: 51 (34.5%) | A: 97 (65.5%) | AA: 31 (41.9%) | CA: 35 (47.3%) | CC: 8 (10.8%) |
| FTD/MND | C: 29 (20.4%) | A: 113 (79.6%) | AA: 45 (63.4%) | CA: 23 (32.4%) | CC: 3 (4.2%) |
| MND | C: 57 (23.8%) | A: 183 (76.3%) | AA: 71 (59.2%) | CA: 41 (34.2%) | CC: 8 (6.7%) |
| Controls | C: 187 (25.0%) | A: 561 (75.0%) | AA: 208 (55.6%) | CA: 145 (38.8%) | CC: 21 (5.6%) |
| rs4630362 (*TXNRD1*) |  |  |  |  |  |
| All Cases | G: 66 (10.0%) | C: 592 (90.0%) | CC: 271 (82.4%) | CG: 50 (15.2%) | GG: 8 (2.4%) |
| Proband cases | G: 47 (8.9%) | C: 481 (91.1%) | CC: 221 (83.7%) | CG: 39 (14.8%) | GG: 4 (1.5%) |
| FTD | G: 10 (6.8%) | C: 138 (93.2%) | CC: 64 (86.5%) | CG: 10 (13.5%) | GG: 0 (0.0%) |
| FTD/MND | G: 15 (10.6%) | C: 127 (89.4%) | CC: 59 (83.1%) | CG: 9 (12.7%) | GG: 3 (4.2%) |
| MND | G: 22 (9.2%) | C: 216 (90.8%) | CC: 98 (82.4%) | CG: 20 (16.8%) | GG: 1 (0.8%) |
| Controls | G: 81 (10.8%) | C: 667 (89.2%) | CC: 300 (80.2%) | CG: 67 (17.9%) | GG: 7 (1.9%) |
| rs4674345 (*CYP27A1*) |  |  |  |  |  |
| All Cases | A: 293 (44.7%) | G: 335 (51.1%) | GG: 89 (27.1%) | GA: 157 (47.9%) | AA: 82 (25%) |
| Proband cases | A: 261 (49.4%) | G: 267 (50.6%) | GG: 69 (26.1%) | GA: 129 (48.9%) | AA: 66 (25%) |
| FTD | A: 79 (54.1%) | G: 67 (45.9%) | GG: 17 (23.3%) | GA: 33 (45.2%) | AA: 23 (31.5%) |
| FTD/MND | A: 68 (47.9%) | G: 74 (52.1%) | GG: 20 (28.2%) | GA: 34 (47.9%) | AA: 17 (23.9%) |
| MND | A: 114 (47.5%) | G: 126 (52.5%) | GG: 32 (26.7%) | GA: 62 (51.7%) | AA: 26 (21.7%) |
| Controls | A: 358 (47.9%) | G: 390 (52.1%) | GG: 102 (27.3%) | GA: 186 (49.7%) | AA: 86 (23%) |
| rs4925 (*GSTO1*) |  |  |  |  |  |
| All Cases | A: 176 (26.7%) | C: 451 (68.3%) | CC: 156 (47.3%) | CA: 139 (42.1%) | AA: 35 (10.6%) |
| Proband cases | A: 162 (30.6%) | C: 368 (69.4%) | CC: 131 (49.4%) | CA: 106 (40%) | AA: 28 (10.6%) |
| FTD | A: 49 (33.1%) | C: 99 (66.9%) | CC: 31 (41.9%) | CA: 37 (50%) | AA: 6 (8.1%) |
| FTD/MND | A: 46 (32.4%) | C: 96 (67.6%) | CC: 33 (46.5%) | CA: 30 (42.3%) | AA: 8 (11.3%) |
| MND | A: 67 (27.9%) | C: 173 (72.1%) | CC: 67 (55.8%) | CA: 39 (32.5%) | AA: 14 (11.7%) |
| Controls | A: 230 (30.8%) | C: 516 (69.2%) | CC: 173 (46.4%) | CA: 170 (45.6%) | AA: 30 (8%) |
| rs5848 (*GRN*) |  |  |  |  |  |
| All Cases | A: 220 (33.3%) | G: 440 (66.7%) | GG: 150 (45.5%) | GA: 140 (42.4%) | AA: 40 (12.1%) |
| Proband cases | A: 158 (29.8%) | G: 372 (70.2%) | GG: 133 (50.2%) | GA: 106 (40.0%) | AA: 26 (9.8%) |
| FTD | A: 53 (35.8%) | G: 95 (64.2%) | GG: 32 (43.2%) | GA: 31 (41.9%) | AA: 11 (14.9%) |
| FTD/MND | A: 40 (28.2%) | G: 102 (71.8%) | GG: 36 (50.7%) | GA: 30 (42.3%) | AA: 5 (7.0%) |
| MND | A: 65 (27.1%) | G: 175 (72.9%) | GG: 65 (54.2%) | GA: 45 (37.5%) | AA: 10 (8.3%) |
| Controls | A: 216 (29.0%) | G: 530 (71.0%) | GG: 183 (49.1%) | GA: 164 (44%) | AA: 26 (7.0%) |
| rs6052771 (*PRNP*) |  |  |  |  |  |
| All Cases | G: 249 (37.8%) | A: 409 (62.2%) | AA: 133 (40.4%) | GA: 143 (43.5%) | GG: 53 (16.1%) |
| Proband cases | G: 211 (40.0%) | A: 317 (60.0%) | AA: 101 (38.3%) | GA: 115 (43.6%) | GG: 48 (18.2%) |
| FTD | G: 52 (35.1%) | A: 96 (64.9%) | AA: 35 (47.3%) | GA: 26 (35.1%) | GG: 13 (17.6%) |
| FTD/MND | G: 68 (47.9%) | A: 74 (52.1%) | AA: 18 (25.4%) | GA: 38 (53.5%) | GG: 15 (21.1%) |
| MND | G: 91 (38.2%) | A: 147 (61.8%) | AA: 48 (40.3%) | GA: 51 (42.9%) | GG: 20 (16.8%) |
| Controls | G: 271 (36.2%) | A: 477 (63.8%) | AA: 158 (42.2%) | GA: 161 (43.0%) | GG: 55 (14.7%) |
| rs6265 (*BDNF*) |  |  |  |  |  |
| All Cases | A: 123 (18.7%) | G: 535 (81.3%) | GG: 214 (65.0%) | GA: 107 (32.5%) | AA: 8 (2.4%) |
| Proband cases | A: 104 (19.7%) | G: 424 (80.3%) | GG: 167 (63.3%) | GA: 90 (34.1%) | AA: 7 (2.7%) |
| FTD | A: 28 (19.2%) | G: 118 (80.8%) | GG: 47 (64.4%) | GA: 24 (32.9%) | AA: 2 (2.7%) |
| FTD/MND | A: 28 (19.7%) | G: 114 (80.3%) | GG: 45 (63.4%) | GA: 24 (33.8%) | AA: 2 (2.8%) |
| MND | A: 48 (20.0%) | G: 192 (80.0%) | GG: 75 (62.5%) | GA: 42 (35.0%) | AA: 3 (2.5%) |
| Controls | A: 152 (20.4%) | G: 594 (79.6%) | GG: 229 (61.4%) | GA: 136 (36.5%) | AA: 8 (2.1%) |
| rs662 (*PON1*) |  |  |  |  |  |
| All Cases | G: 193 (29.3%) | A: 465 (70.7%) | AA: 163 (49.5%) | GA: 139 (42.2%) | GG: 27 (8.2%) |
| Proband cases | G: 150 (28.4%) | A: 378 (71.6%) | AA: 133 (50.4%) | GA: 112 (42.4%) | GG: 19 (7.2%) |
| FTD | G: 47 (32.2%) | A: 99 (67.8%) | AA: 33 (45.2%) | GA: 33 (45.2%) | GG: 7 (9.6%) |
| FTD/MND | G: 35 (24.6%) | A: 107 (75.4%) | AA: 40 (56.3%) | GA: 27 (38.0%) | GG: 4 (5.6%) |
| MND | G: 68 (28.3%) | A: 172 (71.7%) | AA: 60 (50.0%) | GA: 52 (43.3%) | GG: 8 (6.7%) |
| Controls | G: 229 (30.6%) | A: 519 (69.4%) | AA: 187 (50.0%) | GA: 145 (38.8%) | GG: 42 (11.2%) |
| rs6690993 (*FGGY/FLJ0986*) |  |  |  |  |  |
| All Cases | G: 236 (35.8%) | A: 424 (64.2%) | AA: 143 (43.3%) | GA: 138 (41.8%) | GG: 49 (14.8%) |
| Proband cases | G: 194 (36.6%) | A: 336 (63.4%) | AA: 114 (43.0%) | GA: 108 (40.8%) | GG: 43 (16.2%) |
| FTD | G: 47 (31.8%) | A: 101 (68.2%) | AA: 36 (48.6%) | GA: 29 (39.2%) | GG: 9 (12.2%) |
| FTD/MND | G: 60 (42.3%) | A: 82 (57.7%) | AA: 26 (36.6%) | GA: 30 (42.3%) | GG: 15 (21.1%) |
| MND | G: 87 (36.3%) | A: 153 (63.8%) | AA: 52 (43.3%) | GA: 49 (40.8%) | GG: 19 (15.8%) |
| Controls | G: 274 (36.6%) | A: 474 (63.4%) | AA: 142 (38%) | GA: 190 (50.8%) | GG: 42 (11.2%) |
| rs6985069 (*ELP3*) |  |  |  |  |  |
| All Cases | G: 155 (23.5%) | A: 505 (76.5%) | AA: 195 (59.1%) | GA: 115 (34.8%) | GG: 20 (6.1%) |
| Proband cases | G: 125 (23.6%) | A: 405 (76.4%) | AA: 157 (59.2%) | GA: 91 (34.3%) | GG: 17 (6.4%) |
| FTD | G: 35 (23.6%) | A: 113 (76.4%) | AA: 45 (60.8%) | GA: 23 (31.1%) | GG: 6 (8.1%) |
| FTD/MND | G: 33 (23.2%) | A: 109 (76.8%) | AA: 43 (60.6%) | GA: 23 (32.4%) | GG: 5 (7.0%) |
| MND | G: 57 (23.8%) | A: 183 (76.3%) | AA: 69 (57.5%) | GA: 45 (37.5%) | GG: 6 (5.0%) |
| Controls | G: 216 (28.9%) | A: 532 (71.1%) | AA: 190 (50.8%) | GA: 152 (40.6%) | GG: 32 (8.6%) |
| rs699947 (*VEGF*) |  |  |  |  |  |
| All Cases | A: 336 (50.9%) | C: 324 (49.1%) | CC: 75 (22.7%) | CA: 174 (52.7%) | AA: 81 (24.5%) |
| Proband cases | A: 266 (50.2%) | C: 264 (49.8%) | CC: 66 (24.9%) | CA: 132 (49.8%) | AA: 67 (25.3%) |
| FTD | A: 72 (48.6%) | C: 76 (51.4%) | CC: 21 (28.4%) | CA: 34 (45.9%) | AA: 19 (25.7%) |
| FTD/MND | A: 70 (49.3%) | C: 72 (50.7%) | CC: 16 (22.5%) | CA: 40 (56.3%) | AA: 15 (21.1%) |
| MND | A: 124 (51.7%) | C: 116 (48.3%) | CC: 29 (24.2%) | CA: 58 (48.3%) | AA: 33 (27.5%) |
| Controls | A: 351 (47.1%) | C: 395 (52.9%) | CC: 108 (29.0%) | CA: 179 (48.0%) | AA: 86 (23.1%) |
| rs7018487 (*UBAP1*) |  |  |  |  |  |
| All Cases | G: 223 (34.0%) | T: 433 (66.0%) | TT: 146 (44.5%) | GT: 141 (43.0%) | GG: 41 (12.5%) |
| Proband cases | G: 161 (30.6%) | T: 365 (69.4%) | TT: 128 (48.7%) | GT: 109 (41.4%) | GG: 26 (9.9%) |
| FTD | G: 52 (35.6%) | T: 94 (64.4%) | TT: 32 (43.8%) | GT: 30 (41.1%) | GG: 11 (15.1%) |
| FTD/MND | G: 42 (30.0%) | T: 98 (70.0%) | TT: 32 (45.7%) | GT: 34 (48.6%) | GG: 4 (5.7%) |
| MND | G: 67 (27.9%) | T: 173 (72.1%) | TT: 64 (53.3%) | GT: 45 (37.5%) | GG: 11 (9.2%) |
| Controls | G: 255 (34.1%) | T: 493 (65.9%) | TT: 163 (43.6%) | GT: 167 (44.7%) | GG: 44 (11.8%) |
| rs706118 (*BAG1*) |  |  |  |  |  |
| All Cases | G: 143 (21.7%) | T: 515 (78.3%) | TT: 202 (61.4%) | GT: 111 (33.7%) | GG: 16 (4.9%) |
| Proband cases | G: 119 (22.5%) | T: 409 (77.5%) | TT: 156 (59.1%) | GT: 97 (36.7%) | GG: 11 (4.2%) |
| FTD | G: 36 (24.7%) | T: 110 (75.3%) | TT: 41 (56.2%) | GT: 28 (38.4%) | GG: 4 (5.5%) |
| FTD/MND | G: 30 (21.1%) | T: 112 (78.9%) | TT: 44 (62.0%) | GT: 24 (33.8%) | GG: 3 (4.2%) |
| MND | G: 53 (22.1%) | T: 187 (77.9%) | TT: 71 (59.2%) | GT: 45 (37.5%) | GG: 4 (3.3%) |
| Controls | G: 197 (26.3%) | T: 551 (73.7%) | TT: 209 (55.9%) | GT: 133 (35.6%) | GG: 32 (8.6%) |
| rs7403881 (*MT-Ie*) |  |  |  |  |  |
| All Cases | C: 335 (50.8%) | G: 325 (49.2%) | GG: 82 (24.8%) | GC: 161 (48.8%) | CC: 87 (26.4%) |
| Proband cases | C: 266 (50.2%) | G: 264 (49.8%) | GG: 67 (25.3%) | GC: 130 (49.1%) | CC: 68 (25.7%) |
| FTD | C: 76 (51.4%) | G: 72 (48.6%) | GG: 17 (23.0%) | GC: 38 (51.4%) | CC: 19 (25.7%) |
| FTD/MND | C: 72 (50.7%) | G: 70 (49.3%) | GG: 18 (25.4%) | GC: 34 (47.9%) | CC: 19 (26.8%) |
| MND | C: 118 (49.2%) | G: 122 (50.8%) | GG: 32 (26.7%) | GC: 58 (48.3%) | CC: 30 (25.0%) |
| Controls | C: 356 (47.6%) | G: 392 (52.4%) | GG: 110 (29.4%) | GC: 172 (46.0%) | CC: 92 (24.6%) |
| rs7493 (*PON2*) |  |  |  |  |  |
| All Cases | G: 123 (18.7%) | C: 535 (81.3%) | CC: 218 (66.3%) | CG: 99 (30.1%) | GG: 12 (3.6%) |
| Proband cases | G: 106 (20.1%) | C: 422 (79.9%) | CC: 169 (64.0%) | CG: 84 (31.8%) | GG: 11 (4.2%) |
| FTD | G: 28 (18.9%) | C: 120 (81.1%) | CC: 50 (67.6%) | CG: 20 (27.0%) | GG: 4 (5.4%) |
| FTD/MND | G: 36 (25.7%) | C: 104 (74.3%) | CC: 38 (54.3%) | CG: 28 (40.0%) | GG: 4 (5.7%) |
| MND | G: 42 (17.5%) | C: 198 (82.5%) | CC: 81 (67.5%) | CG: 36 (30.0%) | GG: 3 (2.5%) |
| Controls | G: 176 (23.5%) | C: 572 (76.5%) | CC: 219 (58.6%) | CG: 134 (35.8%) | GG: 21 (5.6%) |
| rs9430335 (*TARDBP*) |  |  |  |  |  |
| All Cases | C: 107 (16.2%) | T: 553 (83.8%) | TT: 232 (70.3%) | TC: 89 (27.0%) | CC: 9 (2.7%) |
| Proband cases | C: 88 (16.6%) | T: 442 (83.4%) | TT: 184 (69.4%) | TC: 74 (27.9%) | CC: 7 (2.6%) |
| FTD | C: 21 (14.2%) | T: 127 (85.8%) | TT: 54 (73.0%) | TC: 19 (25.7%) | CC: 1 (1.4%) |
| FTD/MND | C: 22 (15.5%) | T: 120 (84.5%) | TT: 50 (70.4%) | TC: 20 (28.2%) | CC: 1 (1.4%) |
| MND | C: 45 (18.8%) | T: 195 (81.3%) | TT: 80 (66.7%) | TC: 35 (29.2%) | CC: 5 (4.2%) |
| Controls | C: 121 (16.2%) | T: 625 (83.8%) | TT: 261 (70.0%) | TC: 103 (27.6%) | CC: 9 (2.4%) |
| Allele (*APOE*) |  |  |  |  |  |
| All Cases | E4+: 112 (15.1%) | E4-: 632 (84.9%) | E4-E4-: 269 (72.3%) | E4-E4+: 94 (25.3%) | E4+E4+: 9 (2.4%) |
| Proband cases | E4+: 99 (18.8%) | E4-: 429 (81.3%) | E4-E4-: 175 (66.3%) | E4-E4+: 79 (29.9%) | E4+E4+: 10 (3.8%) |
| FTD | E4+: 23 (15.8%) | E4-: 123 (84.2%) | E4-E4-: 52 (71.2%) | E4-E4+: 19 (26.0%) | E4+E4+: 2 (2.7%) |
| FTD/MND | E4+: 31 (21.8%) | E4-: 111 (78.2%) | E4-E4-: 43 (60.6%) | E4-E4+: 25 (35.2%) | E4+E4+: 3 (4.2%) |
| MND | E4+: 45 (18.8%) | E4-: 195 (81.3%) | E4-E4-: 80 (66.7%) | E4-E4+: 35 (29.2%) | E4+E4+: 5 (4.2%) |
| Controls | E4+: 120 (18.3%) | E4-: 536 (81.7%) | E4-E4-: 220 (67.1%) | E4-E4+: 96 (29.3%) | E4+E4+: 12 (3.7%) |
| Haplotype (*MAPT*) |  |  |  |  |  |
| All Cases | H2: 135 (20.5%) | H1: 525 (79.5%) | H1H1: 212 (64.2%) | H1H2: 101 (30.6%) | H2H2: 17 (5.2%) |
| Proband cases | H2: 105 (19.8%) | H1: 425 (80.2%) | H1H1: 173 (65.3%) | H1H2: 79 (29.8%) | H2H2: 13 (4.9%) |
| FTD | H2: 25 (16.9%) | H1: 123 (83.1%) | H1H1: 54 (73.0%) | H1H2: 15 (20.3%) | H2H2: 5 (6.8%) |
| FTD/MND | H2: 21 (14.8%) | H1: 121 (85.2%) | H1H1: 51 (71.8%) | H1H2: 19 (26.8%) | H2H2: 1 (1.4%) |
| MND | H2: 59 (24.6%) | H1: 181 (75.4%) | H1H1: 68 (56.7%) | H1H2: 45 (37.5%) | H2H2: 7 (5.8%) |
| Controls | H2: 175 (23.4%) | H1: 573 (76.6%) | H1H1: 222 (59.4%) | H1H2: 129 (34.5%) | H2H2: 23 (6.1%) |
|  |  |  |  |  |  |

**Table S2 Associations with age at onset under additive, dominant and recessive models in the overall group of FTD, FTD/MND, and MND probands (n = 243)**

|  |  |  | Additive model | | Dominant model | | Recessive model | |
| --- | --- | --- | --- | --- | --- | --- | --- | --- |
| Variant (gene) | MA | MAF | Regression coefficient (95% CI) | P-value | Regression coefficient (95% CI) | P-value | Regression coefficient (95% CI) | P-value |
| rs10260404 (*DPP6*) | C | 35.7% | -1.43 (-3.10, 0.25) | 0.094 | -1.01 (-3.35, 1.34) | 0.40 | -3.69 (-7.05, -0.33) | 0.031 |
| rs10487132 (*PON3*) | G | 39.1% | -0.06 (-1.65, 1.53) | 0.94 | -0.46 (-2.85, 1.94) | 0.71 | 0.49 (-2.49, 3.47) | 0.75 |
| rs1052352 (*FUS*) | C | 45.7% | -0.85 (-2.42, 0.73) | 0.29 | -1.34 (-3.83, 1.15) | 0.29 | -0.94 (-3.70, 1.83) | 0.50 |
| rs10971977 (*UBAP1*) | T | 48.1% | 1.71 (0.10, 3.32) | 0.038 | 0.97 (-1.63, 3.56) | 0.46 | 3.77 (1.08, 6.46) | 0.006 |
| rs1130409 (*APEX1*) | G | 44.8% | -1.04 (-2.70, 0.61) | 0.22 | -2.53 (-5.04, -0.01) | 0.049 | 0.13 (-2.79, 3.05) | 0.93 |
| rs11701 (*ANG*) | G | 13.3% | N/A | N/A | -0.57 (-3.19, 2.04) | 0.67 | N/A | N/A |
| rs12608932 (*UNC13A*) | C | 37.2% | -0.54 (-2.10, 1.03) | 0.50 | 0.15 (-2.19, 2.50) | 0.90 | -2.27 (-5.30, 0.75) | 0.14 |
| rs13268953 (*ELP3*) | G | 44.4% | 0.43 (-1.21, 2.08) | 0.60 | 0.85 (-1.65, 3.34) | 0.50 | 0.21 (-2.72, 3.14) | 0.89 |
| rs1541160 (*KIFAP3*) | G | 27.8% | -1.70 (-3.53, 0.14) | 0.071 | -2.10 (-4.41, 0.21) | 0.074 | -2.05 (-6.47, 2.36) | 0.36 |
| rs165932 (*PS-1*) | C | 44.4% | -0.44 (-2.16, 1.27) | 0.61 | -1.05 (-3.60, 1.50) | 0.42 | 0.08 (-2.94, 3.11) | 0.96 |
| rs17350674 (*KIF24*) | A | 20.0% | N/A | N/A | 1.53 (-0.86, 3.93) | 0.21 | N/A | N/A |
| rs1799945 (*HFE*) | G | 11.2% | N/A | N/A | 2.39 (-0.39, 5.18) | 0.092 | N/A | N/A |
| rs1799983 (*NOS3*) | T | 34.0% | 0.01 (-1.80, 1.82) | 0.99 | -0.17 (-2.51, 2.18) | 0.89 | 0.51 (-3.45, 4.47) | 0.80 |
| rs1800435 (*ALAD*) | C | 8.2% | N/A | N/A | -1.42 (-4.65, 1.81) | 0.39 | N/A | N/A |
| rs1801133 (*MTHFR*) | T | 35.6% | -0.16 (-1.82, 1.50) | 0.85 | -0.50 (-2.84, 1.83) | 0.67 | 0.37 (-2.96, 3.70) | 0.83 |
| rs2010963 (*VEGF*) | C | 32.7% | -0.30 (-1.99, 1.39) | 0.72 | -1.41 (-3.73, 0.90) | 0.23 | 1.97 (-1.57, 5.52) | 0.27 |
| rs2279720 (*CHMP2B*) | A | 6.6% | N/A | N/A | 1.83 (-1.58, 5.25) | 0.29 | N/A | N/A |
| rs2306677 (*ITPR2*) | T | 8.6% | N/A | N/A | 0.36 (-2.83, 3.55) | 0.82 | N/A | N/A |
| rs407135 (*SLC11A2*) | C | 25.1% | -0.07 (-1.93, 1.79) | 0.94 | 0.03 (-2.34, 2.40) | 0.98 | -0.52 (-4.95, 3.90) | 0.82 |
| rs4630362 (*TXNRD1*) | G | 8.9% | N/A | N/A | -0.29 (-3.40, 2.83) | 0.86 | N/A | N/A |
| rs4674345 (*CYP27A1*) | A | 48.6% | 0.43 (-1.18, 2.03) | 0.60 | 1.15 (-1.43, 3.73) | 0.38 | -0.05 (-2.74, 2.63) | 0.97 |
| rs4925 (*GSTO1*) | A | 29.8% | 0.32 (-1.43, 2.06) | 0.72 | 0.22 (-2.10, 2.54) | 0.85 | 0.96 (-2.93, 4.84) | 0.63 |
| rs5848 (*GRN*) | A | 29.4% | -1.24 (-2.99, 0.51) | 0.16 | -2.89 (-5.18, -0.60) | 0.014 | 2.08 (-1.83, 5.99) | 0.30 |
| rs6052771 (*PRNP*) | G | 40.5% | 2.13 (0.54, 3.71) | 0.009 | 1.88 (-0.55, 4.32) | 0.13 | 4.42 (1.51, 7.32) | 0.003* |
| rs6265 (*BDNF*) | A | 20.2% | N/A | N/A | -0.67 (-3.08, 1.74) | 0.59 | N/A | N/A |
| rs662 (*PON1*) | G | 27.7% | -0.97 (-2.85, 0.90) | 0.31 | -2.37 (-4.69, -0.04) | 0.046 | 3.14 (-1.37, 7.65) | 0.17 |
| rs6690993 (*FGGY/FLJ0986*) | G | 37.0% | -0.58 (-2.18, 1.01) | 0.47 | 0.20 (-2.15, 2.54) | 0.87 | -2.54 (-5.63, 0.55) | 0.11 |
| rs6985069 (*ELP3*) | G | 23.7% | -0.02 (-1.89, 1.84) | 0.98 | -0.52 (-2.88, 1.83) | 0.66 | 1.91 (-2.75, 6.57) | 0.42 |
| rs699947 (*VEGF*) | A | 49.6% | -0.68 (-2.30, 0.93) | 0.41 | -2.01 (-4.63, 0.62) | 0.13 | 0.19 (-2.47, 2.86) | 0.89 |
| rs7018487 (*UBAP1*) | G | 29.5% | -2.62 (-4.36, -0.89) | 0.003* | -3.06 (-5.35, -0.78) | 0.009 | -4.27 (-8.19, -0.35) | 0.033 |
| rs706118 (*BAG1*) | G | 22.3% | 1.83 (-0.17, 3.83) | 0.072 | 2.23 (-0.11, 4.56) | 0.062 | 1.69 (-4.11, 7.48) | 0.57 |
| rs7403881 (*MT-Ie*) | C | 49.0% | 2.09 (0.49, 3.69) | 0.011 | 3.95 (1.36, 6.54) | 0.003* | 1.61 (-1.06, 4.29) | 0.24 |
| rs7493 (*PON2*) | G | 20.5% | -0.37 (-2.42, 1.69) | 0.73 | -0.44 (-2.87, 1.99) | 0.72 | -0.41 (-6.24, 5.41) | 0.89 |
| rs9430335 (*TARDBP*) | C | 17.3% | N/A | N/A | 0.64 (-1.87, 3.15) | 0.62 | N/A | N/A |
| Allele (*APOE*) | E4 | 18.8% | N/A | N/A | -0.52 (-2.95, 1.92) | 0.68 | N/A | N/A |
| Haplotype (*MAPT*) | H2 | 19.8% | -0.78 (-2.77, 1.21) | 0.44 | -1.07 (-3.55, 1.41) | 0.40 | -0.59 (-5.74, 4.55) | 0.82 |
| MA = minor allele; MAF = minor allele frequency; CI = confidence interval. The association between each variant and age at disease onset was evaluated using a linear regression model adjusted for gender and disease group (FTD, FTD/MND, or MND). Additive models (effect of each additional minor allele), dominant models (presence versus absence of the minor allele), and recessive models (presence versus absence of two copies of the minor allele) were utilized. Regression coefficients are interpreted as the change in mean age at onset for each additional minor allele (additive models), presence of the minor allele (dominant models), or presence of two copies of the minor allele (recessive models). N/A is given for both the regression coefficient and p-value in additive and recessive models if there were fewer than ten individuals with the rare genotype. *Indicates a significant association with disease using a false discovery rate (FDR) of 10%. In age at onset association analysis for all FTD, FTD/MND, and MND probands, p-values ≤ 0.0034 were considered as statistically significant. | | | | | | | | |

**Table S3a Associations with survival after onset under additive, dominant and recessive models in the overall group of FTD, FTD/MND, and MND probands (n = 221)**

|  |  |  | Additive model | | Dominant model | | Recessive model | |
| --- | --- | --- | --- | --- | --- | --- | --- | --- |
| Variant (gene) | MA | MAF | RR (95% CI) | P-value | RR (95% CI) | P-value | RR (95% CI) | P-value |
| rs10260404 (*DPP6*) | C | 36.4% | 0.89 (0.66, 1.21) | 0.46 | 0.86 (0.56, 1.34) | 0.51 | 0.85 (0.48, 1.53) | 0.59 |
| rs10487132 (*PON3*) | G | 39.6% | 0.72 (0.53, 0.96) | 0.024 | 0.63 (0.41, 0.97) | 0.035 | 0.61 (0.34, 1.08) | 0.092 |
| rs1052352 (*FUS*) | C | 45.5% | 0.90 (0.68, 1.19) | 0.44 | 1.02 (0.65, 1.60) | 0.93 | 0.68 (0.40, 1.16) | 0.16 |
| rs10971977 (*UBAP1*) | T | 47.3% | 0.84 (0.63, 1.12) | 0.24 | 0.73 (0.46, 1.15) | 0.17 | 0.87 (0.54, 1.40) | 0.56 |
| rs1130409 (*APEX1*) | G | 43.9% | 0.91 (0.68, 1.23) | 0.55 | 0.85 (0.54, 1.33) | 0.48 | 0.94 (0.56, 1.56) | 0.80 |
| rs11701 (*ANG*) | G | 13.2% | N/A | N/A | 1.09 (0.68, 1.76) | 0.72 | N/A | N/A |
| rs12608932 (*UNC13A*) | C | 36.9% | 1.36 (1.03, 1.80) | 0.033 | 1.33 (0.87, 2.03) | 0.19 | 1.88 (1.13, 3.10) | 0.014 |
| rs13268953 (*ELP3*) | G | 44.1% | 1.47 (1.04, 2.08) | 0.028 | 1.30 (0.81, 2.06) | 0.27 | 2.20 (1.25, 3.88) | 0.006 |
| rs1541160 (*KIFAP3*) | G | 27.8% | 1.01 (0.72, 1.42) | 0.95 | 0.98 (0.64, 1.49) | 0.92 | 1.15 (0.52, 2.54) | 0.72 |
| rs165932 (*PS-1*) | C | 43.9% | 0.89 (0.63, 1.25) | 0.49 | 0.92 (0.58, 1.48) | 0.74 | 0.78 (0.42, 1.45) | 0.43 |
| rs17350674 (*KIF24*) | A | 20.8% | N/A | N/A | 1.37 (0.90, 2.10) | 0.15 | N/A | N/A |
| rs1799945 (*HFE*) | G | 11.6% | N/A | N/A | 0.90 (0.54, 1.49) | 0.68 | N/A | N/A |
| rs1799983 (*NOS3*) | T | 33.9% | 1.00 (0.72, 1.39) | 1.00 | 0.99 (0.65, 1.52) | 0.97 | 1.03 (0.48, 2.23) | 0.94 |
| rs1800435 (*ALAD*) | C | 7.7% | N/A | N/A | 1.26 (0.66, 2.42) | 0.49 | N/A | N/A |
| rs1801133 (*MTHFR*) | T | 35.5% | 0.85 (0.64, 1.13) | 0.26 | 0.89 (0.59, 1.35) | 0.57 | 0.62 (0.33, 1.18) | 0.15 |
| rs2010963 (*VEGF*) | C | 32.6% | 0.89 (0.64, 1.24) | 0.51 | 0.77 (0.50, 1.17) | 0.21 | 1.26 (0.64, 2.48) | 0.50 |
| rs2279720 (*CHMP2B*) | A | 7.0% | N/A | N/A | 1.08 (0.53, 2.20) | 0.82 | N/A | N/A |
| rs2306677 (*ITPR2*) | T | 8.1% | N/A | N/A | 0.91 (0.48, 1.69) | 0.76 | N/A | N/A |
| rs407135 (*SLC11A2*) | C | 25.3% | 1.22 (0.85, 1.74) | 0.28 | 1.27 (0.82, 1.95) | 0.28 | 1.24 (0.49, 3.15) | 0.65 |
| rs4630362 (*TXNRD1*) | G | 9.3% | N/A | N/A | 1.69 (0.96, 2.97) | 0.071 | N/A | N/A |
| rs4674345 (*CYP27A1*) | A | 47.0% | 0.95 (0.72, 1.26) | 0.73 | 1.08 (0.68, 1.71) | 0.74 | 0.79 (0.48, 1.30) | 0.35 |
| rs4925 (*GSTO1*) | A | 29.6% | 0.67 (0.47, 0.96) | 0.027 | 0.58 (0.38, 0.89) | 0.010 | 0.79 (0.34, 1.84) | 0.58 |
| rs5848 (*GRN*) | A | 28.1% | 1.64 (1.22, 2.22) | 0.001* | 1.84 (1.20, 2.84) | 0.005 | 2.25 (1.22, 4.15) | 0.009 |
| rs6052771 (*PRNP*) | G | 40.7% | 1.08 (0.83, 1.40) | 0.57 | 1.38 (0.87, 2.20) | 0.17 | 0.90 (0.56, 1.45) | 0.66 |
| rs6265 (*BDNF*) | A | 19.5% | N/A | N/A | 0.79 (0.51, 1.22) | 0.28 | N/A | N/A |
| rs662 (*PON1*) | G | 25.9% | 1.18 (0.82, 1.69) | 0.37 | 1.07 (0.69, 1.66) | 0.76 | 2.06 (0.93, 4.56) | 0.076 |
| rs6690993 (*FGGY/FLJ0986*) | G | 36.2% | 0.92 (0.68, 1.25) | 0.61 | 0.83 (0.55, 1.26) | 0.38 | 1.08 (0.59, 1.96) | 0.81 |
| rs6985069 (*ELP3*) | G | 24.0% | 1.52 (1.09, 2.13) | 0.014 | 1.81 (1.12, 2.90) | 0.015 | 1.78 (0.81, 3.93) | 0.15 |
| rs699947 (*VEGF*) | A | 48.6% | 0.91 (0.67, 1.24) | 0.55 | 0.92 (0.55, 1.53) | 0.74 | 0.86 (0.52, 1.41) | 0.54 |
| rs7018487 (*UBAP1*) | G | 30.1% | 1.18 (0.86, 1.62) | 0.31 | 1.22 (0.79, 1.86) | 0.37 | 1.28 (0.65, 2.52) | 0.47 |
| rs706118 (*BAG1*) | G | 22.3% | N/A | N/A | 1.44 (0.93, 2.23) | 0.11 | N/A | N/A |
| rs7403881 (*MT-Ie*) | C | 48.2% | 1.20 (0.90, 1.60) | 0.22 | 1.30 (0.79, 2.14) | 0.30 | 1.29 (0.80, 2.07) | 0.29 |
| rs7493 (*PON2*) | G | 19.5% | N/A | N/A | 1.22 (0.79, 1.87) | 0.37 | N/A | N/A |
| rs9430335 (*TARDBP*) | C | 17.0% | N/A | N/A | 1.84 (1.15, 2.94) | 0.011 | N/A | N/A |
| Allele (*APOE*) | E4 | 19.1% | N/A | N/A | 0.97 (0.61, 1.54) | 0.90 | N/A | N/A |
| Haplotype (*MAPT*) | H2 | 19.0% | 1.07 (0.74, 1.55) | 0.73 | 1.12 (0.70, 1.77) | 0.65 | 0.96 (0.35, 2.67) | 0.94 |
| MA = minor allele; MAF = minor allele frequency; RR = relative risk; CI = confidence interval. The association between each variant and survival after onset was evaluated using a Cox proportional hazards regression model adjusted for age at onset, gender, and disease group (FTD, FTD/MND, or MND). Additive models (effect of each additional minor allele), dominant models (presence versus absence of the minor allele), and recessive models (presence versus absence of two copies of the minor allele) were utilized. N/A is given for both the relative risk and p-value in additive and recessive models if there were fewer than ten individuals with the rare genotype. *Indicates a significant association with disease using a false discovery rate (FDR) of 10%. In survival after onset association analysis for all FTD, FTD/MND, and MND probands, p-values ≤ 0.0024 were considered as statistically significant. | | | | | | | | |

**Table S3b Associations with survival after onset under additive, dominant and recessive models in FTD probands (n = 58)**

|  |  |  | Additive model | | Dominant model | | Recessive model | |
| --- | --- | --- | --- | --- | --- | --- | --- | --- |
| Variant (gene) | MA | MAF | RR (95% CI) | P-value | RR (95% CI) | P-value | RR (95% CI) | P-value |
| rs10260404 (*DPP6*) | C | 33.3% | N/A | N/A | 0.81 (0.40, 1.62) | 0.55 | N/A | N/A |
| rs10487132 (*PON3*) | G | 43.1% | 1.04 (0.68, 1.59) | 0.85 | 1.04 (0.54, 2.03) | 0.90 | 1.09 (0.50, 2.41) | 0.83 |
| rs1052352 (*FUS*) | C | 44.0% | 0.72 (0.46, 1.14) | 0.16 | 0.68 (0.33, 1.38) | 0.28 | 0.61 (0.27, 1.35) | 0.22 |
| rs10971977 (*UBAP1*) | T | 44.0% | 0.93 (0.6, 1.46) | 0.76 | 0.96 (0.48, 1.92) | 0.91 | 0.84 (0.36, 1.93) | 0.67 |
| rs1130409 (*APEX1*) | G | 45.7% | 1.09 (0.69, 1.72) | 0.73 | 1.04 (0.50, 2.18) | 0.91 | 1.21 (0.56, 2.60) | 0.63 |
| rs11701 (*ANG*) | G | 13.2% | N/A | N/A | 0.78 (0.34, 1.79) | 0.56 | N/A | N/A |
| rs12608932 (*UNC13A*) | C | 31.0% | N/A | N/A | 0.96 (0.50, 1.84) | 0.89 | N/A | N/A |
| rs13268953 (*ELP3*) | G | 45.7% | 1.90 (1.09, 3.32) | 0.023 | 1.49 (0.67, 3.31) | 0.33 | 3.65 (1.56, 8.55) | 0.003* |
| rs1541160 (*KIFAP3*) | G | 23.3% | N/A | N/A | 0.77 (0.38, 1.57) | 0.47 | N/A | N/A |
| rs165932 (*PS-1*) | C | 44.0% | 0.55 (0.33, 0.94) | 0.029 | 0.42 (0.19, 0.91) | 0.029 | 0.58 (0.26, 1.30) | 0.19 |
| rs17350674 (*KIF24*) | A | 19.8% | N/A | N/A | 1.30 (0.67, 2.52) | 0.44 | N/A | N/A |
| rs1799945 (*HFE*) | G | 9.6% | N/A | N/A | 1.07 (0.50, 2.32) | 0.86 | N/A | N/A |
| rs1799983 (*NOS3*) | T | 32.8% | N/A | N/A | 0.82 (0.42, 1.62) | 0.57 | N/A | N/A |
| rs1800435 (*ALAD*) | C | 6.0% | N/A | N/A | N/A | N/A | N/A | N/A |
| rs1801133 (*MTHFR*) | T | 31.0% | N/A | N/A | 0.49 (0.23, 1.02) | 0.055 | N/A | N/A |
| rs2010963 (*VEGF*) | C | 37.9% | N/A | N/A | 0.67 (0.35, 1.27) | 0.22 | N/A | N/A |
| rs2279720 (*CHMP2B*) | A | 6.0% | N/A | N/A | N/A | N/A | N/A | N/A |
| rs2306677 (*ITPR2*) | T | 11.4% | N/A | N/A | 0.78 (0.38, 1.63) | 0.51 | N/A | N/A |
| rs407135 (*SLC11A2*) | C | 34.5% | N/A | N/A | 1.78 (0.87, 3.65) | 0.12 | N/A | N/A |
| rs4630362 (*TXNRD1*) | G | 7.8% | N/A | N/A | N/A | N/A | N/A | N/A |
| rs4674345 (*CYP27A1*) | A | 51.8% | 0.84 (0.54, 1.29) | 0.42 | 1.01 (0.48, 2.10) | 0.99 | 0.56 (0.24, 1.29) | 0.17 |
| rs4925 (*GSTO1*) | A | 31.0% | N/A | N/A | 0.66 (0.34, 1.28) | 0.22 | N/A | N/A |
| rs5848 (*GRN*) | A | 33.6% | N/A | N/A | 1.99 (1.02, 3.88) | 0.045 | N/A | N/A |
| rs6052771 (*PRNP*) | G | 36.2% | 1.26 (0.85, 1.86) | 0.26 | 1.68 (0.85, 3.32) | 0.14 | 1.19 (0.56, 2.51) | 0.65 |
| rs6265 (*BDNF*) | A | 20.2% | N/A | N/A | 0.64 (0.31, 1.34) | 0.24 | N/A | N/A |
| rs662 (*PON1*) | G | 27.2% | N/A | N/A | 0.95 (0.47, 1.90) | 0.87 | N/A | N/A |
| rs6690993 (*FGGY/FLJ0986*) | G | 28.4% | N/A | N/A | 0.72 (0.37, 1.39) | 0.32 | N/A | N/A |
| rs6985069 (*ELP3*) | G | 26.7% | N/A | N/A | 1.62 (0.82, 3.22) | 0.17 | N/A | N/A |
| rs699947 (*VEGF*) | A | 47.4% | 1.07 (0.69, 1.65) | 0.77 | 1.18 (0.57, 2.43) | 0.66 | 1.01 (0.47, 2.16) | 0.98 |
| rs7018487 (*UBAP1*) | G | 36.8% | N/A | N/A | 1.06 (0.50, 2.22) | 0.89 | N/A | N/A |
| rs706118 (*BAG1*) | G | 25.4% | N/A | N/A | 0.92 (0.47, 1.78) | 0.80 | N/A | N/A |
| rs7403881 (*MT-Ie*) | C | 50.9% | 2.82 (1.51, 5.26) | 0.001* | 2.80 (0.93, 8.44) | 0.068 | 3.81 (1.71, 8.46) | 0.001* |
| rs7493 (*PON2*) | G | 16.4% | N/A | N/A | 0.82 (0.40, 1.68) | 0.59 | N/A | N/A |
| rs9430335 (*TARDBP*) | C | 13.8% | N/A | N/A | 1.31 (0.61, 2.80) | 0.48 | N/A | N/A |
| Allele (*APOE*) | E4 | 14.9% | N/A | N/A | 3.13 (1.45, 6.74) | 0.004* | N/A | N/A |
| Haplotype (*MAPT*) | H2 | 12.9% | N/A | N/A | 1.68 (0.77, 3.66) | 0.19 | N/A | N/A |
| MA = minor allele; MAF = minor allele frequency; RR = relative risk; CI = confidence interval. The association between each variant and survival after onset was evaluated using a Cox proportional hazards regression model adjusted for age at onset and gender. Additive models (effect of each additional minor allele), dominant models (presence versus absence of the minor allele), and recessive models (presence versus absence of two copies of the minor allele) were utilized. N/A is given for both the relative risk and p-value in additive and recessive models if there were fewer than ten individuals with the rare genotype. *Indicates a significant association with disease using a false discovery rate (FDR) of 10%. In survival after onset association analysis for FTD probands, p-values ≤ 0.0075 were considered as statistically significant. | | | | | | | | |

**Table S3c Associations with survival after onset under additive, dominant and recessive models in MND probands (n = 107)**

|  |  |  | Additive model | | Dominant model | | Recessive model | |
| --- | --- | --- | --- | --- | --- | --- | --- | --- |
| Variant (gene) | MA | MAF | RR (95% CI) | P-value | RR (95% CI) | P-value | RR (95% CI) | P-value |
| rs10260404 (*DPP6*) | C | 38.8% | 1.32 (0.54, 3.26) | 0.54 | 1.74 (0.46, 6.65) | 0.42 | 1.00 (0.19, 5.23) | 1.00 |
| rs10487132 (*PON3*) | G | 43.0% | 0.69 (0.30, 1.60) | 0.38 | 0.54 (0.18, 1.65) | 0.28 | 0.83 (0.17, 4.02) | 0.82 |
| rs1052352 (*FUS*) | C | 47.2% | 0.98 (0.48, 2.01) | 0.97 | 1.39 (0.37, 5.19) | 0.62 | 0.70 (0.19, 2.54) | 0.58 |
| rs10971977 (*UBAP1*) | T | 46.7% | 1.02 (0.48, 2.18) | 0.96 | 1.05 (0.33, 3.36) | 0.94 | 1.00 (0.25, 4.02) | 1.00 |
| rs1130409 (*APEX1*) | G | 41.1% | 0.42 (0.15, 1.23) | 0.12 | 0.33 (0.10, 1.16) | 0.085 | 0.58 (0.06, 5.33) | 0.63 |
| rs11701 (*ANG*) | G | 13.2% | N/A | N/A | 0.77 (0.22, 2.76) | 0.69 | N/A | N/A |
| rs12608932 (*UNC13A*) | C | 40.2% | 2.87 (1.38, 5.97) | 0.003* | 3.67 (0.99, 13.58) | 0.051 | 5.65 (1.82, 17.58) | 0.003* |
| rs13268953 (*ELP3*) | G | 42.1% | N/A | N/A | 0.96 (0.32, 2.94) | 0.95 | N/A | N/A |
| rs1541160 (*KIFAP3*) | G | 31.3% | 2.01 (0.84, 4.78) | 0.11 | 1.70 (0.50, 5.75) | 0.39 | 4.00 (0.96, 16.64) | 0.057 |
| rs165932 (*PS-1*) | C | 48.1% | 0.41 (0.18, 0.93) | 0.032 | 0.39 (0.13, 1.22) | 0.11 | 0.16 (0.02, 1.27) | 0.083 |
| rs17350674 (*KIF24*) | A | 22.0% | 0.72 (0.31, 1.66) | 0.44 | 0.70 (0.22, 2.27) | 0.55 | 0.41 (0.05, 3.49) | 0.42 |
| rs1799945 (*HFE*) | G | 12.1% | N/A | N/A | 5.37 (1.61, 17.96) | 0.006 | N/A | N/A |
| rs1799983 (*NOS3*) | T | 35.0% | 0.86 (0.35, 2.07) | 0.73 | 0.46 (0.15, 1.40) | 0.17 | 2.51 (0.67, 9.47) | 0.17 |
| rs1800435 (*ALAD*) | C | 9.3% | N/A | N/A | 0.0% versus 15.9% | 0.003* | N/A | N/A |
| rs1801133 (*MTHFR*) | T | 37.9% | 1.21 (0.55, 2.67) | 0.63 | 1.56 (0.48, 5.04) | 0.46 | 0.93 (0.19, 4.58) | 0.93 |
| rs2010963 (*VEGF*) | C | 33.2% | 0.98 (0.43, 2.23) | 0.95 | 0.82 (0.28, 2.44) | 0.73 | 1.42 (0.30, 6.67) | 0.66 |
| rs2279720 (*CHMP2B*) | A | 8.4% | N/A | N/A | 1.17 (0.26, 5.36) | 0.84 | N/A | N/A |
| rs2306677 (*ITPR2*) | T | 9.1% | N/A | N/A | 1.44 (0.30, 6.78) | 0.65 | N/A | N/A |
| rs407135 (*SLC11A2*) | C | 25.2% | N/A | N/A | 0.90 (0.29, 2.76) | 0.85 | N/A | N/A |
| rs4630362 (*TXNRD1*) | G | 9.3% | N/A | N/A | 5.46 (1.39, 21.51) | 0.015 | N/A | N/A |
| rs4674345 (*CYP27A1*) | A | 45.3% | 1.07 (0.50, 2.32) | 0.86 | 1.40 (0.41, 4.80) | 0.59 | 0.78 (0.17, 3.51) | 0.74 |
| rs4925 (*GSTO1*) | A | 28.5% | 0.67 (0.27, 1.67) | 0.39 | 0.61 (0.17, 2.14) | 0.44 | 0.49 (0.06, 4.03) | 0.51 |
| rs5848 (*GRN*) | A | 24.8% | N/A | N/A | 2.21 (0.73, 6.73) | 0.16 | N/A | N/A |
| rs6052771 (*PRNP*) | G | 38.2% | 1.00 (0.50, 2.02) | 1.00 | 1.26 (0.39, 4.07) | 0.70 | 0.76 (0.20, 2.85) | 0.68 |
| rs6265 (*BDNF*) | A | 18.7% | N/A | N/A | 1.86 (0.65, 5.35) | 0.25 | N/A | N/A |
| rs662 (*PON1*) | G | 26.2% | N/A | N/A | 1.06 (0.36, 3.19) | 0.91 | N/A | N/A |
| rs6690993 (*FGGY/FLJ0986*) | G | 34.6% | 0.61 (0.23, 1.63) | 0.32 | 0.33 (0.10, 1.13) | 0.078 | 2.40 (0.50, 11.64) | 0.28 |
| rs6985069 (*ELP3*) | G | 24.8% | N/A | N/A | 0.88 (0.29, 2.69) | 0.82 | N/A | N/A |
| rs699947 (*VEGF*) | A | 50.5% | 1.30 (0.60, 2.81) | 0.51 | 1.59 (0.42, 6.05) | 0.50 | 1.28 (0.38, 4.38) | 0.69 |
| rs7018487 (*UBAP1*) | G | 28.5% | 1.54 (0.67, 3.54) | 0.31 | 1.87 (0.61, 5.80) | 0.28 | 1.46 (0.26, 8.21) | 0.67 |
| rs706118 (*BAG1*) | G | 21.0% | N/A | N/A | 1.34 (0.46, 3.93) | 0.59 | N/A | N/A |
| rs7403881 (*MT-Ie*) | C | 46.3% | 0.97 (0.45, 2.12) | 0.94 | 1.78 (0.47, 6.74) | 0.39 | 0.33 (0.04, 2.57) | 0.29 |
| rs7493 (*PON2*) | G | 16.8% | N/A | N/A | 0.64 (0.19, 2.16) | 0.47 | N/A | N/A |
| rs9430335 (*TARDBP*) | C | 19.2% | N/A | N/A | 1.51 (0.51, 4.46) | 0.45 | N/A | N/A |
| Allele (*APOE*) | E4 | 20.1% | N/A | N/A | 0.34 (0.08, 1.54) | 0.16 | N/A | N/A |
| Haplotype (*MAPT*) | H2 | 24.8% | N/A | N/A | 0.94 (0.30, 2.97) | 0.92 | N/A | N/A |
| MA = minor allele; MAF = minor allele frequency; RR = relative risk; CI = confidence interval. The association between each variant and survival after onset was evaluated using a Cox proportional hazards regression model adjusted for age at onset and gender. Additive models (effect of each additional minor allele), dominant models (presence versus absence of the minor allele), and recessive models (presence versus absence of two copies of the minor allele) were utilized. For rs1800435:G>C, none of the 19 MND patients (0.0%) who were carriers of the minor allele died compared to 14 of 78 MND patients (15.9%) who were not carriers of the minor allele; due to the zero cell count, Cox regression analysis was not possible, and the p-value of 0.003 results from a log-rank test. N/A is given for both the relative risk and p-value in additive and recessive models if there were fewer than ten individuals with the rare genotype. *Indicates a significant association with disease using a false discovery rate (FDR) of 10%. In survival after onset association analysis for MND probands, p-values ≤ 0.0041 were considered as statistically significant. | | | | | | | | |

**Table S4 Combinations of *ELP3* variants rs13268953 and rs6985069 in relation to survival after onset in the overall group**

| rs13268953 genotype | rs6985069 genotype | Number of patients | Relative risk (95% CI) | P-value |  |
| --- | --- | --- | --- | --- | --- |
|  |  |  | Test of overall difference: P=0.001 | |  |
| AA or AG | AA | 99 | 1.00 (reference) | N/A | Test of interaction: P=0.33 |
| AA or AG | AG or GG | 80 | 2.25 (1.33, 3.79) | 0.003 |  |
| GG | AA | 30 | 2.93 (1.54, 5.55) | 0.001 |  |
| GG | AG or GG | 12 | 3.70 (0.86, 16.01) | 0.080 |  |
